# Molecular signatures of post-traumatic stress disorder in war-zone-exposed veteran and active-duty soldiers

**DOI:** 10.1016/j.xcrm.2023.101045

**Published:** 2023-05-16

**Authors:** Seid Muhie, Aarti Gautam, Ruoting Yang, Burook Misganaw, Bernie J. Daigle, Synthia H. Mellon, Janine D. Flory, Duna Abu-Amara, Inyoul Lee, Kai Wang, Ryan Rampersaud, PTSD Systems Biology Consortium, Leroy Hood, Rachel Yehuda, Charles R. Marmar, Owen M. Wolkowitz, Kerry J. Ressler, Francis J. Doyle, Rasha Hammamieh, Marti Jett

**Affiliations:** 1 Medical Readiness Systems Biology, Walter Reed Army Institute of Research, Silver Spring, MD 20910, USA; 2 The Geneva Foundation, Silver Spring, MD 20910, USA; 3 Vysnova Inc., Landover, MD 20785, USA; 4 Departments of Biological Sciences and Computer Science, The University of Memphis, Memphis, TN 38152, USA; 5 Department of Obstetrics, Gynecology & Reproductive Sciences, University of California, San Francisco, San Francisco, CA 94143, USA; 6 Office of Mental Health, James J. Peters VA Medical Center, Bronx, NY 10468, USA; 7 Department of Psychiatry, Icahn School of Medicine at Mount Sinai, New York, NY 10468, USA; 8 Department of Psychiatry, New York University Grossman School of Medicine, New York, NY 10016, USA; 9 Institute for Systems Biology, Seattle, WA 98109, USA; 10 Department of Psychiatry and Behavioral Sciences, University of California, San Francisco, San Francisco, CA 94143, USA; 11 McLean Hospital, Belmont, MA 02478, USA; 12 Harvard Medical School, Boston, MA 02115, USA; 13 Harvard John A. Paulson School of Engineering and Applied Sciences, Harvard University, Cambridge, MA 02134, USA; 14 US Army Medical Research and Development Command, HQ, Walter Reed Army Institute of Research, Silver Spring, MD 20910, USA

**Keywords:** post-traumatic stress disorder, veterans, active duty, multi-omics, molecular signature, oxidative stress, inflammatory response, angiogenesis, metabolic dysregulation, wound healing

## Abstract

Post-traumatic stress disorder (PTSD) is a multisystem syndrome. Integration of systems-level multi-modal datasets can provide a molecular understanding of PTSD. Proteomic, metabolomic, and epigenomic assays are conducted on blood samples of two cohorts of well-characterized PTSD cases and controls: 340 veterans and 180 active-duty soldiers. All participants had been deployed to Iraq and/or Afghanistan and exposed to military-service-related criterion A trauma. Molecular signatures are identified from a discovery cohort of 218 veterans (109/109 PTSD+/−). Identified molecular signatures are tested in 122 separate veterans (62/60 PTSD+/−) and in 180 active-duty soldiers (PTSD+/−). Molecular profiles are computationally integrated with upstream regulators (genetic/methylation/microRNAs) and functional units (mRNAs/proteins/metabolites). Reproducible molecular features of PTSD are identified, including activated inflammation, oxidative stress, metabolic dysregulation, and impaired angiogenesis. These processes may play a role in psychiatric and physical comorbidities, including impaired repair/wound healing mechanisms and cardiovascular, metabolic, and psychiatric diseases.

## Highlights

• Genetic, epigenetic, proteomic, and metabolomic signatures of PTSD are identified
• Degrees of alterations of signatures correlate with severity and chronicity of symptoms
• Inflammation, oxidative stress, and metabolic dysfunction implicated in tissue injury
• Tissue injury and impaired healing may contribute to the course and persistence of PTSD

## Graphical abstract

**Figure:**
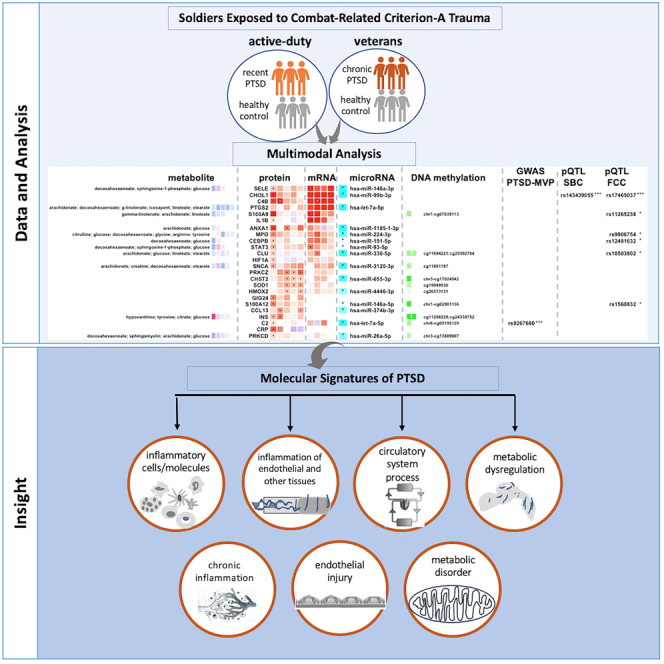

## Teaser

Muhie et al. identify epigenetic, proteomic, and metabolomic signatures of combat-related PTSD. Alteration degrees correlate with severity and chronicity of symptoms. Inflammation, oxidative stress, metabolic dysfunction, and inhibited angiogenesis are implicated in tissue injury. Impaired healing of injured tissues may contribute to the course and persistence of PTSD and comorbidities.

## Introduction

Post-traumatic stress disorder (PTSD) affects more than 12% of combat-exposed soldiers and about 6% of the US population.^1^ PTSD, in addition to being a stress response with intrusive memories, avoidance of reminders, emotional numbing, negative beliefs, and hyperarousal, can progress to a multisystem syndrome with comorbidities. Despite its prevalence and socio-economic burden, molecular mediators of onset, course, and persistence of PTSD are not yet fully established. Particularly, molecular signatures along its temporal and severity trajectories are essential for better understanding of the molecular of PTSD pathogenesis. This includes systems-level identification of molecular alterations that underlie PTSD and its comorbidities that can be profiled in circulating cellular and molecular entities. Even though systemic responses vary by organ system, cellular processes contributing to PTSD-related syndromes are shown to be associated with circulating molecular species.

For example, alterations in circulating molecular features have been implicated in mitochondrial dysfunction and inflammation,^2^^,^^3^^,^^4^^,^^5^^,^^6^^,^^7^^,^^8^ delayed wound healing,^9^^,^^10^^,^^11^ cardiovascular diseases,^12^^,^^13^^,^^14^^,^^15^ metabolic disorders,^2^^,^^7^^,^^16^^,^^17^ type 2 diabetes mellitus (T2DM),^18^ chronic pain,^19^^,^^20^ oxidative stress associated with inflammation and endothelial cell dysfunction,^21^ immune modulation,^22^^,^^23^ glucocorticoid receptor sensitivity,^3^ epigenetic aging,^24^ neurotrophic activity,^25^ and disease progression,^26^ including biomarker panels to facilitate screening for PTSD diagnosis.^8^^,^^27^

Even with increased ongoing efforts, previous studies were constrained by lack of adequate study cohorts at different chronicity and severity levels of the disease, multi-modal genome-wide datasets encompassing the full spectrum of molecular species (genetic, epigenetic, transcriptomics, proteomic, and metabolomics), and/or computational integration of these datasets. For example, most prior studies were conducted either on veterans with chronic PTSD or on more diverse civilian participants and/or with limited multi-modal molecular datasets.

The present study comprises much larger proteomic data (SomaLogic) along with genome-wide DNA methylation, microRNA, and metabolomics datasets assayed on blood samples collected from two well-characterized cohorts. Cohorts included 340 veterans (300 males and 40 females) with chronic PTSD and 180 active-duty soldiers (159 males and 21 females) including a subset of service members with subclinical PTSD and a longitudinal group of service members assessed pre- and post-deployment with recent-onset PTSD symptoms.

Compositions of cohorts and multi-omics assays were designed to identify altered molecular signatures of the temporal and severity trajectories of PTSD. First, the male cohorts were evaluated for biomolecular signatures that correlated with clinical features of PTSD severity and chronicity. Then findings from the male cohorts of veterans and active-duty soldiers were qualitatively compared with the smaller subgroups of female veterans and active-duty service members. Molecular signatures from these cohorts were also compared with published postmortem gene expression data from brain subregions of PTSD cases and controls^28^ and prior large-scale PTSD genome-wide association studies (GWAS).^29^^,^^30^

Overall, this study combined data-driven discovery approaches with prior mechanistic insights of PTSD pathogenesis. Findings presented here are not discrete collections of altered molecular features and signaling pathways but rather a set of coherent molecular events interconnected across temporal and severity steps of PTSD and its comorbidities. Differentially altered pathways, in conjunction with pre-existing genetic and epigenetic factors, are likely to mediate PTSD-related syndromes and seem to contribute to the course, severity, and persistence of the disorder.

## Results

### Cohort composition

**Figure 1:**
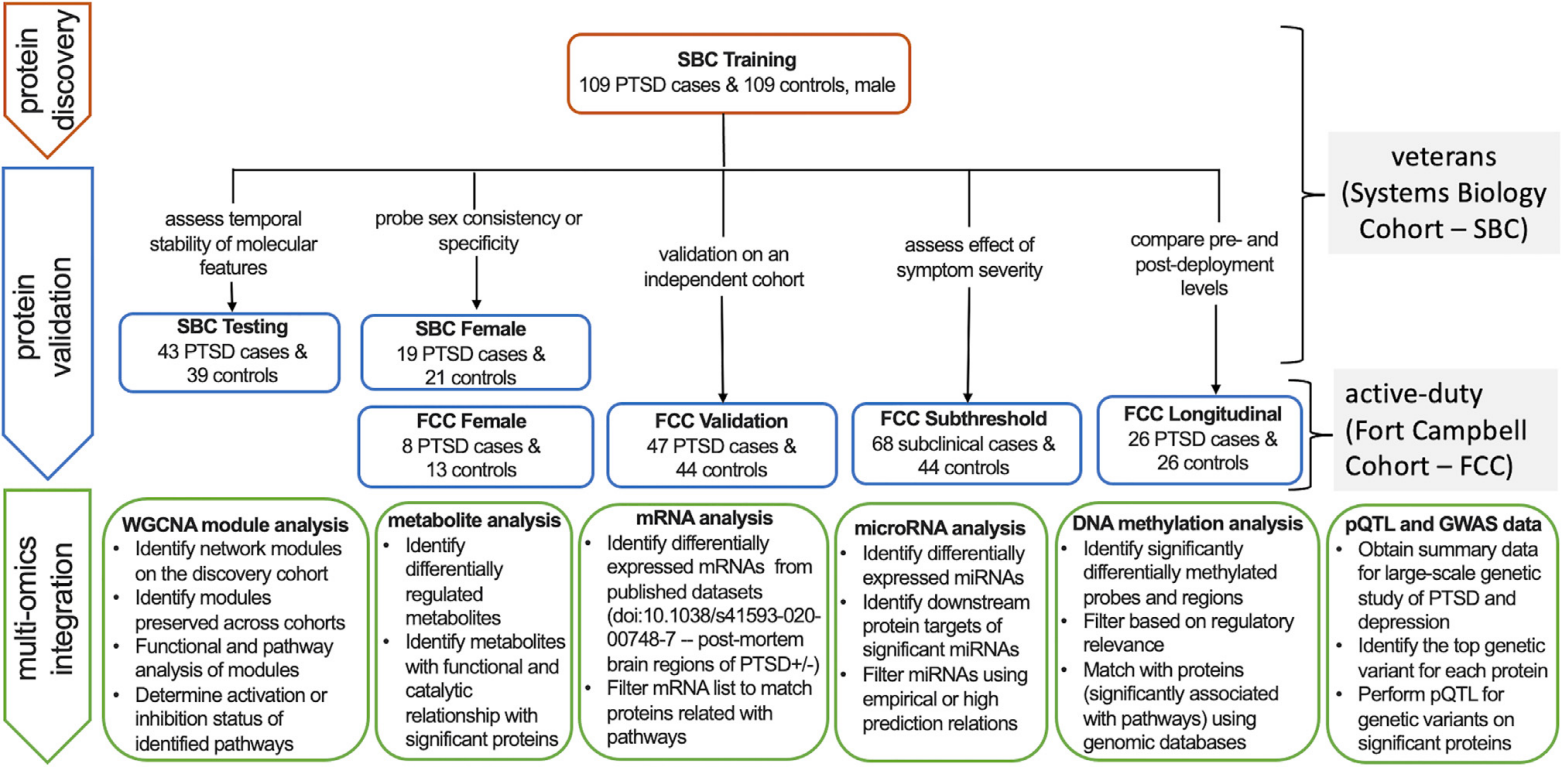
Overall workflow of the study Identifying, validating, and characterizing of PTSD-associated proteins and integration with multi-modal molecular features from blood samples of veterans and active-duty military participants, published postmortem brain regions, and summary statistics from publicly available genome-wide association studies. PTSD cases and trauma-exposed healthy controls composed of two well-characterized cohorts: Systems Biology Consortium (SBC: 340 veterans) and Fort Campbell Cohort (FCC: 180 active-duty service members). All participants had been deployed to Iraq and/or Afghanistan. The active-duty cohort included blood biomarkers and clinical features assessed longitudinally before and after deployment. Male veterans with chronic PTSD (CAPS scores ≥ 40; ≥3 months duration) and matched controls (CAPS total score < 20) were recruited into training and testing cohorts. A smaller, case-control female veteran cohort with chronic PTSD was recruited with the same inclusion criteria. Active-duty males with recent PTSD (PCL ≥ 38; 3 days before or 90–180 days post-deployment), active-duty males with subthreshold recent PTSD (PCL ≥ 22 to < 38; 3 days before or 90–180 post-deployment), and active-duty females with recent PTSD; all cohorts included matched controls (PCL < 22). Recent onset PTSD case-controls (n = 26) were a longitudinal male active-duty cohort with recent PTSD (n = 26 controls: PCL < 22 at 2 weeks pre-deployment; n = 26 cases: PCL ≥ 31 at 3 days before or 90–180 days post-deployment). The FCC Validation and FCC Subthreshold groups shared the same controls. Comparability of molecular datasets across cohorts was verified by quality control output graphs presented in Figures S1 and S2. CAPS: Clinician-Administered PTSD Scale; PCL: PTSD Checklist.

The Systems Biology Consortium (SBC) and Fort Campbell Cohort (FCC) cohorts were composed of 340 veterans (300 males and 40 females) and 180 active-duty service members (159 males and 21 females) respectively (Figure 1). Twenty-six members of the active-duty group were followed longitudinally for an average of 13 ± 0.75 months. All participants (those with and without PTSD) were exposed to military-service-related PTSD criterion A events.

Participants were evaluated for psychiatric symptoms, work and relationship functioning, and neurocognitive functioning including measures of attention and emotion regulation. Comprehensive biomarkers were ascertained from whole blood, plasma, serum, and buffy-coat samples (Table 1). Routine clinical labs were collected including complete blood count, lipid panel, inflammatory markers, and liver functioning tests.^27^^,^^31^ Molecular profiles associated with PTSD-related clinical features were identified in a “training” dataset of 218 SBC male veterans (SBC Training: 109/109 PTSD+/−) (Figure 1) and tested in a newly recruited SBC Testing group of 82 male veterans (SBC Testing: 43/39 PTSD+/−). Earlier molecular trajectories of identified molecular and pathway signatures were then validated in an independent cohort of 180 FCC external validation (159 males and 21 females) active-duty soldiers.

**Table 1:** Input samples and platform for each of the multi-omics assays

Omics assay	Input sample (blood fraction)	Platform/kit (supplemental materials and methods)
Proteome	serum	appropriate blood fraction for SomaScan (SomaLogic) proteomics platform
Metabolome	plasma	blood fraction appropriate for and assayed using Metabolon metabolomics platform
MicroRNAs (miRs)	exosomal miRs (exosomes isolated from plasma)	exosomal miRs assayed using Illumina’s small RNA-Seq kit (exosomal miRs are more relevant blood compartment in interpreting PTSD molecular signatures as they are more likely to cross into the brain)
DNA methylome	whole blood genomic DNA (isolated from PAXgene blood DNA tubes)	the whole blood genomic DNA was bisulfite-converted using the EZ96 DNA methylation kit (Zymo Research), and the bisulfite-treated DNA was assayed using Infinium HumanMethylation450 BeadChip (genome-wide DNA methylation array from Illumina)

Note: during downstream normalization and analyses, appropriate covariates and potential confounders were assessed and corrected/accounted for. The table summarizing blood tubes and samples for molecular assays is given with the supplemental material (Table S8).

### Molecular indicators of PTSD

**Figure 2:**
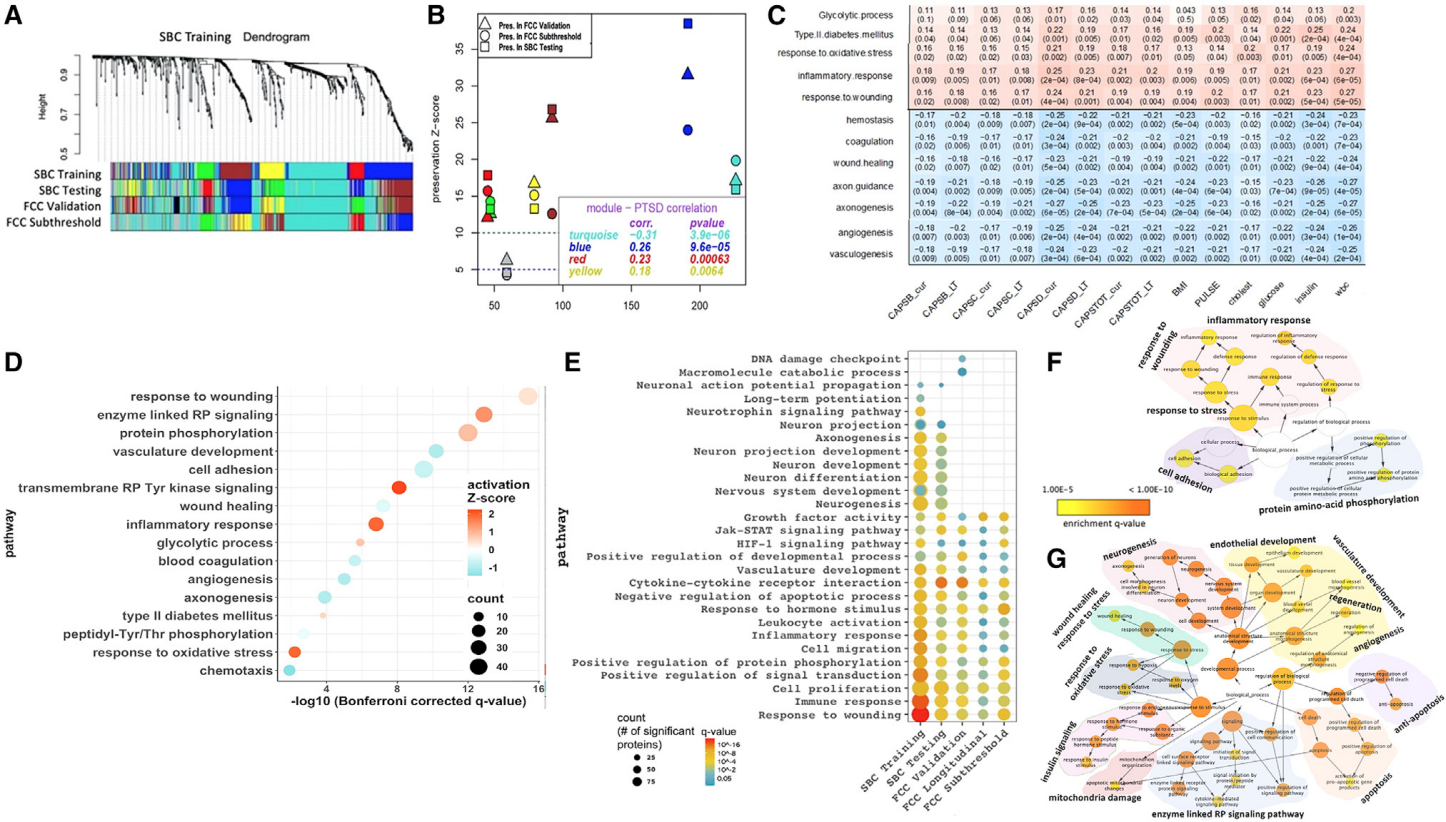
Modular networks and enriched pathways across cohorts (A) Identification of protein co-expression modules associated with PTSD by weighted gene co-expression network analysis (WGCNA). Module identification by hierarchical clustering tree (dendrogram) of the consensus network comprising 1,305 proteins where branches of the dendrogram grouped together densely interconnected, highly co-expressed proteins. Modules were identified in the SBC Training group (109/109 PTSD+/−), shown in the first band underneath the tree. Colors represent each modular network. Subsequent bands indicate modules in the SBC Testing (43/39 PTSD+/−), FCC Validation (47/44 PTSD+/−), and FCC Subthreshold (68/44 PTSD subclinical/controls) groups. (B) Module preservation identified six highly preserved modules (preservation *Z* score > 10; ≥30 proteins per module). Four modules (turquoise, yellow, blue, and red) were significantly correlated (p < 0.01) with PTSD across the SBC Testing, FCC Validation, and FCC Subthreshold (Figure S3). (C) Biological processes and pathways identified using hypergeometric enrichment filter at q < 0.05, Bonferroni correction (family-wise error rate) followed by pathway activation analyses. Ranking is based on pathway enrichment significance. (D) Pathway or process significantly associated (FDR [false discovery rate]-corrected) with differentially altered proteins (PTSD cases vs. controls) in the SBC Training, SBC Testing, FCC Validation, FCC Longitudinal, and FCC Subthreshold. (E) Significantly activated or inhibited pathways that were correlated with CAPS total current (SBC cohort) and PCL scores (FCC cohort). Top panel, positive correlation (red gradient); bottom panel, negative correlation (blue gradient). (F and G) Pathways and biological processes significantly associated with member proteins of the methylation-protein consensus modules (RP: receptor protein; arrows indicate Gene Ontology (GO) or pathway hierarchy from stem to leaves; background colors show the different classes of pathways). Bubble plots denote q values (red, activated; blue, inhibited), where size corresponds with number of proteins. Complete data are in Figure S4. CAPS: Clinically Administered PTSD Scale; CAPSTOT_Curr: CAPS current total; CAPSTOT_lt: CAPS lifetime total; CAPSB: CAPS criterion B (re-experiencing); CAPSC: criterion C (avoidance of trauma reminders); CAPSD: CAPS criterion D (negative cognitions and affect).

A total of 1,305 proteins were assayed in serum samples from all participants using the SomaLogic platform (Figures S1–S3 and Table S1). Weighted gene correlation network analysis (WGCNA) identified six co-expressed modular networks in the SBC Training cohort that were highly preserved across SBC Training, SBC Testing, FCC External Validation, and FCC Subthreshold-PTSD groups (Figures 2A and 2B and Table S1). Four of the six modular networks were associated with re-experiencing, avoidance, and hyperarousal criteria of PTSD as assessed by the Clinician-Administered PTSD Scale (CAPS)^32^^,^^33^ (Figures 2C and S3B). These pathway differences between PTSD cases and controls persisted after adjusting for BMI, age, ancestry, self-reported race, smoking/cotinine, mild TBI, BDI total, education, and sample collection/processing batches. (Datasets from female participants were analyzed separately due to sample size and sex difference considerations including potential confounding from unmeasured hormonal drug use or menstrual cycle.)

### Consensus WGCNA network analyses

Four of the six identified molecular networks (modules) (Figures 2 and S4 and Table S1) were significantly associated with pathways significantly correlated with clinical features of PTSD (Figures 2D and 2E). Enriched pathways included inflammation, response to oxidative stress, e.g., upregulation of positive regulators (HIF1), downregulation of negative regulators of reactive oxygen species (ROS; SOD2 and CAT), response to hormones and growth factors, and advanced glycation endpoints/receptor for advanced glycation endpoints (AGE/RAGE) signaling and glycolytic processes (ENO1) (Figure S4 and Tables S1 and S2). Parallel to identification of modular networks, PTSD cases vs. controls comparisons were done to identify significant proteins after adjusting for potential confounders (Figure S3A). Common proteins among lists of significant proteins and nodes of highly preserved (and PTSD-correlated) modules were used for downstream pathway enrichments and pathway activation analysis.

Also, from DNA methylation datasets (focusing on *cis*-regulatory sites, probes within 1,500 bp of the promoter regions), we identified 10 modular networks. Four of the 10 modular networks were significantly correlated with PTSD (Figures S3C –S3F). Particularly, two modular networks were (1) highly preserved in the male veterans and moderately preserved in male active-duty soldiers and (2) significantly correlated with symptom clusters of PTSD (Figure S3C). Using methylation datasets for probes within the more proximal promoter regions (within 250 bp from transcription start sites) and the protein data (for the corresponding proteins), we identified three consensus modular networks and significantly associated pathways that are matching with significant pathways identified from protein-based modular networks (Figures 2F and 2G and S3H).

### Pathway enrichment and activation analysis

Response to wounding (*Z* score = 0.41, q value < 3.98E-16) was the most significantly enriched pathway overall (Figures 2D and 2E). Significantly enriched and activated pathways included inflammatory response (*Z* score = 1.9, q value < 1.42E-7), protein metabolism (*Z* score = 1.4, q value < 1.42E-12), apoptosis (*Z* score = 0.92, q < 4.0E-4), and response to oxidative stress (activation *Z* score = 1.93, q < 6.4E-3). Inhibited pathways included wound healing (*Z* score = −0.43, q value < 6.31E-8), essentially normal upkeep/repair pathways such as vasculature development (*Z* score = −1.0, q < 1E-10), angiogenesis (*Z* score = −1, q < 1E-5), and hemostasis (coagulation) (*Z* score = −0.74, q value < 2.51E-6).

Inflammation-related immune responses were consistently activated across cohorts to include pathways involved in inflammatory responses, leukocyte activation, and cell migration with enrichment significant decreasing from veterans to active-duty participants with subclinical PTSD (in parallel to chronicity and severity) (Figure 2E). Glycolytic processes and T2DM pathways were also significantly activated (Figure 2). Pathways associated with vascular development (angiogenesis) were significantly inhibited across cohorts with greater inhibition in chronic PTSD cases in the veterans compared with the more recent onset PTSD cases in the active-duty participants (Figure 2E). Similar results were seen in the female veteran group as in the male veteran groups (Figure S5A). Pathways associated with neurogenesis, neural development, and related pathways were highly inhibited in the veterans but not significant in active-duty groups (Figures 2E and S5A).

These pathways were also identified from enrichment analyses of molecular nodes of the three consensus modular networks constructed using methylation datasets of the promoter regions and protein datasets (Figures 2F and 2G). Two of the three consensus networks were largely associated with stress response and inflammation-related pathways (Figure 2F), and nodes of the third network were significantly associated with response to wounding, oxidative stress, neurogenesis, angiogenesis/vasculature development, insulin signaling, apoptosis, and mitochondrial damage (Figure 2G).

### Association of altered molecules and pathways with PTSD symptom progression, severity, and chronicity

We analyzed changes in multi-modal molecular and pathway signatures in relation to PTSD symptom severity and chronicity in the veterans and active-duty cohorts. The participants in the veteran cohorts included individuals who were diagnosed with chronic PTSD, while the active-duty FCC included participants who were diagnosed with more recent onset PTSD (13 months ± 3 weeks), which were also subdivided into those with higher and lower PTSD Checklist (PCL) values, reflecting different levels of severity of PTSD symptoms.

**Figure 3:**
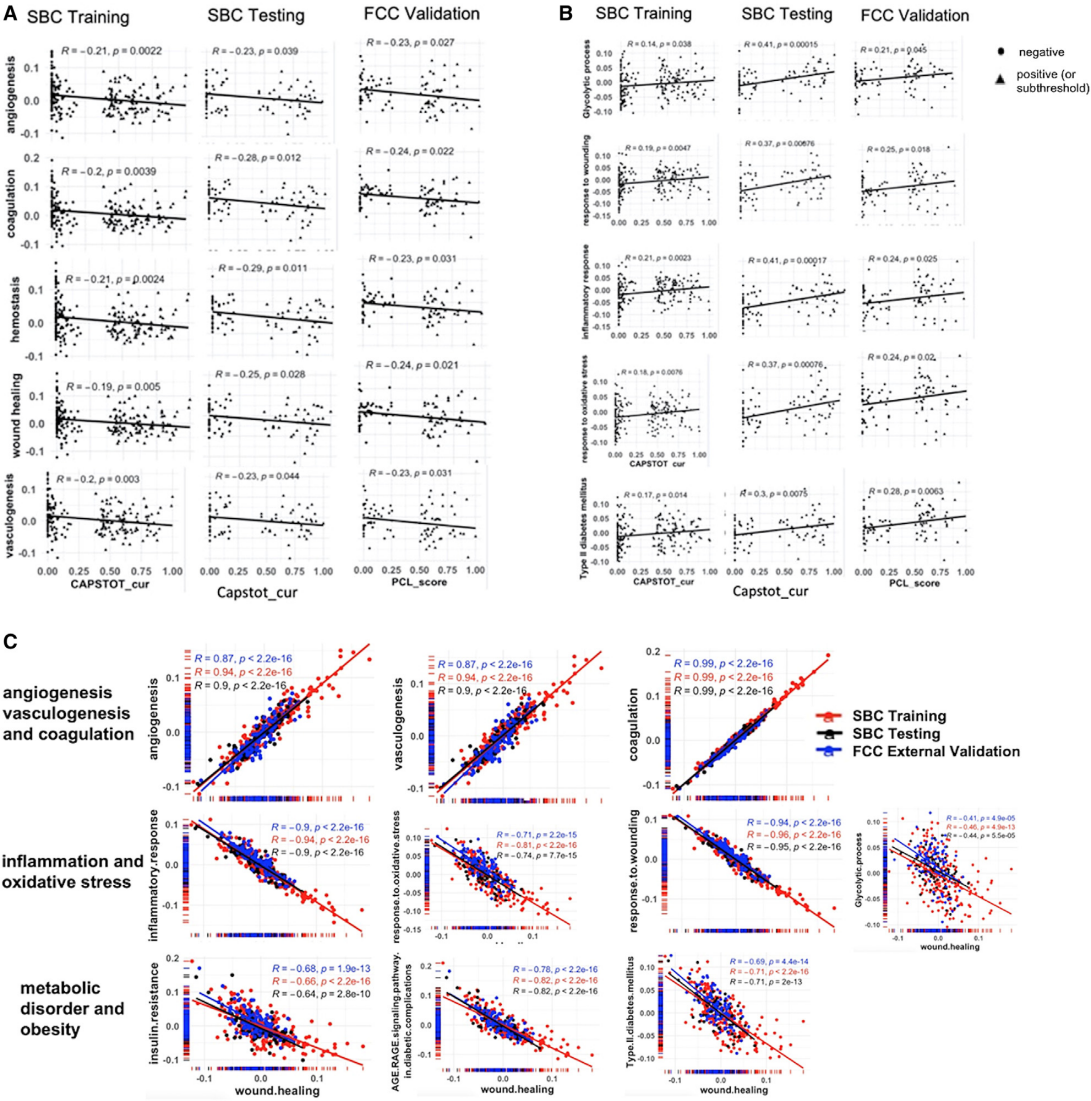
Correlations among significantly inhibited or activated pathways and PTSD diagnostic variables (A) Significant pathways negatively correlated with CAPS total current (SBC cohort) or PCL scores (FCC cohort). (B) Significant pathways positively correlated with CAPS total current (SBC cohort) or PCL scores (FCC cohort). (C) Correlations between significantly inhibited or activated pathways with wound healing in SBC Training (109/109 PTSD+/−), SBC Testing (43/39 PTSD+/−), and FCC Validating (47/44 PTSD+/−) cohorts. Correlations between wound healing and pathways associated with vasculature (top), inflammation/oxidative stress (middle), and metabolic disorder/obesity (bottom) were evaluated in the SBC Training, SBC Testing, and FCC Validation cohorts. PCL, PTSD Checklist, CAPSTOT_cur: the total current score for Clinically Administered PTSD Scale.

Correlations were determined between significantly enriched pathways and clinical features of PTSD (Figures 2C and S6). Pathways associated with angiogenesis, inflammation, oxidative stress, metabolism, and response to wounding were correlated with PTSD symptom severity and chronicity as defined by CAPS total and PCL total scores (Figure 3). Angiogenesis was negatively correlated with CAPS total and PCL total scores across the SBC Training, SBC Testing, FCC External Validation, and FCC Subthreshold-PTSD participants, though to a different extent (Figure 3A). Molecular and pathway alterations associated with inflammatory response, oxidative stress, metabolic dysregulation, and response to wounding were positively correlated with changes in PTSD symptom severity and chronicity as defined by CAPS total and PCL total scores (Figure 3B). PTSD symptom clusters (CAPSB, re-experiencing; CAPSC, avoidance; and CAPSD, hyperarousal) were negatively correlated with angiogenesis/vasculature morphogenesis, epithelization, and coagulation (Figures 2C and S6).

### Altered molecular pathways and markers identified in the longitudinal participants

Molecular pathways significantly associated with impaired angiogenesis, activated inflammation, insulin resistance, mitochondrial dysfunction, decreased bioenergetics, and ROS were consistently altered in the longitudinal group of active-duty participants, though to a lesser extent (Figure 2E).

### Correlations among pathways

Strong positive correlations were observed between wound healing and angiogenesis or coagulation/hemostasis pathways and negative correlations of wound healing with pathways related to inflammation, oxidative stress, and metabolic disorders in the SBC Training, SBC Testing, and FCC External Validation groups (Figure 3C).

### Metabolic dysregulations associated with PTSD

Pathways indicative of metabolic dysregulation such as those associated with insulin resistance, T2DM, reduced bioenergetics, and mitochondrial dysfunction pathways (Figure 2 and Tables S2 and S3) were activated in PTSD cases compared with controls in the SBC Training, SBC Testing, and FCC External Validation groups. Specifically, proteins and metabolites significantly associated with AGE-RAGE signaling (T2DM/inflammation), insulin resistance, impaired glycolytic processes, decreased cellular energy production, and mitochondrial dysfunction were increased in PTSD cases compared with warzone-exposed healthy controls though with decreasing extent of alterations in going from chronic to recent onset and subclinical participants (Figures 2E and 2G and Tables S2 and S3).

### Multi-omics integration

**Figure 4:**
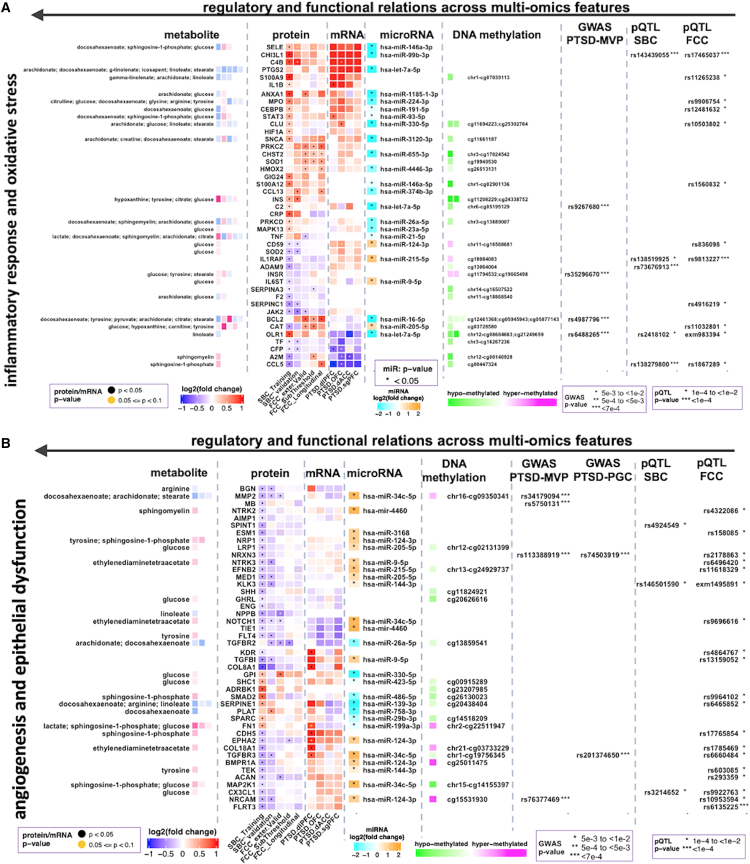
Integrated multi-omics showing regulatory and functional relations (horizontally from right to left) across genetic variants, epigenetic marks, microRNAs, mRNAs proteins, and metabolites (A and B) Differentially expressed proteins (DEPs) that were persistent across PTSD cohorts and associated with (A) activated inflammatory response or oxidative stress, (B) impaired angiogenesis, epithelial dysfunction, or cardiovascular function were integrated with multi-omics datasets and compared across SBC Training (109/109 PTSD+/−), SBC Testing (43/39 PTSD+/−), FCC Validation (47/44 PTSD+/−), FCC Longitudinal (26/26 PTSD+/−), and FCC Subthreshold (68/44 PTSD subclinical/controls) cohorts. Vertical lanes of the protein heatmap correspond to the fold changes of each protein from each group of cohorts (as shown by the labels). SBC: Systems Biology Consortium (veteran cohort), FCC: Fort Campbell (active-duty) Cohort; for brain regions (postmortem mRNA data): dIPFC, dorsolateral pre-frontal cortex (PFC); ACC, anterior cingulate cortex (ACC); dACC, dorsal ACC; sgPFC, subgenual PFC; OFC, orbito-frontal cortex.

Regulatory or functional relations among molecular features from the different omics datasets were used as a basis for the multi-omics integration. Multi-omics analyses approaches were used to evaluate extent of cross-cohort consistency of regulatory and functional relationships among quantitative trait loci, genetic variants, *cis*-regulatory sites (differentially methylated promoter regions [DMRs]), miRNAs, mRNAs, proteins, and metabolites that were associated with the top enriched pathways (Figures 4, S5B, and S8).

Multi-omics datasets comparing PTSD cases and controls in the veteran and active-duty cohorts converged on interconnected pathways: activated inflammation, metabolic dysregulation, increased oxidative stress, impaired angiogenesis/vasculature development, epithelial dysfunction, and response to wounding including patterns of impaired wound healing (Figures 2D and S5A) in line with the temporal and severity stages of PTSD (Figure 2E). Altered molecules from other modalities that showed regulatory and functional relations with significant proteins were corroborative of these observations. Significantly and persistently altered proteins and the corresponding metabolites also were largely consistent with differential methylation of upstream *cis*-regulatory sites and expression status of microRNAs and mRNAs (Figure 4).

Upstream regulatory elements of proteins (and metabolites) associated with inflammatory responses, oxidative stress, and metabolic dysregulation were related with significant minor alleles (SNPs), hypo-methylated DMRs, and downregulated miRs; whereas proteins (and metabolites) that were potentially under the regulatory control of these elements were largely upregulated (Figure 4A). Other important minor alleles, largely hyper-methylated DMRs and upregulated miRs were identified as potential regulatory partners of proteins (and metabolites) associated with inhibited pathways: wound healing, endothelial functions, and vasculature development (angiogenesis) (Figure 4B).

Many of the proteins associated with vasculature development (angiogenesis) and heart development showed expression patterns consistent with impaired wound healing and epithelial dysfunction (Figure 4B). Metabolites such as sphingosine-1-phosphate and lactic acid were also associated with inhibited vasculature development (Figure 4B). Circulating levels of glucose and decreased levels of polyunsaturated fatty acids, omega-3 fatty acids, and essential fatty acids were associated with proteins and DMRs that were implicated in metabolic dysregulation and vasculature development pathways (Figure 4).

### Multi-omics analysis in female veterans and active-duty participants: Qualitative comparisons with findings from male cohorts

Pathway-level changes in veteran and active-duty male PTSD groups were generally also observed in veteran and active-duty female groups. The top significant pathways consistently differentiated in PTSD cases from controls in both male and female veterans and active-duty service members, though fewer pathways were identified in the active-duty females (Figure S5A).

Overall, the multi-omics data corroborated findings from the protein data, including identification of impaired angiogenesis, activated inflammatory response, oxidative stress, metabolic dysregulation, epithelial dysfunction, and wound healing as among the significantly altered pathways in PTSD cases compared with controls (Figures 2, 3, and 4).

### Postmortem gene expression datasets from brain regions of PTSD cases and controls

We used published expression data from brain subregions of PTSD cases and controls^28^ to fill the regulatory gap between significant proteins and significantly altered epigenetic marks. Protein signatures significantly associated with important pathways were found to be largely consistent with significant transcripts profiled from brain subregions implicated in PTSD (Figure 4). Similarity in expression patterns of significant proteins and transcripts from the postmortem expression data corroborate the regulatory connections between the epigenetic layers and the enriched pathways as well as the similarity of the responses between the central and the peripheral systems at the molecular level.

### Relevance of significant proteins in prior large-scale genetic studies

We assessed the enrichment/relevance of significant proteins (belonging to altered pathways) using summary statistics from GWAS of Million Veteran Program (MVP)^29^ for two PTSD phenotypes: quantitative total PCL score and dichotomous PTSD case-control status. Thirty-seven unique proteins belonging to the PTSD-relevant modular networks were found to contain suggestively significant (P < 4E-04) genetic variants associated with PCL score (Table S5). Proteins with the top significant variants include PIK3CG (rs11773880, p = 8.61E-08), NCAM1 (rs2298527, p = 3.44E-07), and GRB2 (rs4789182, p = 7.60E-06), which are important in wound healing, inflammatory response, and neurogenesis; CAMK1D (rs113990432, p = 2.58E-06) activates the transcription factor CREB1 in promoting basal dendritic growth of hippocampal neurons and regulates granulocyte function/respiratory burst; KIRREL3 (rs552640, 2.36E-05) is involved in neurological/cognitive disorders; C3 (rs2241391, p = 2.59E-05) is involved in inflammatory response and synaptic pruning; ROBO2 (rs62268946, p = 3.0E-05) is important in axon guidance and rhythmic processes; HS6ST1 (rs34800061, 3.26E-05) is involved in vasculature development/angiogenesis; MB (rs5750131, 3.48E-05) is important in response to oxidative stress, glucose/energy metabolism, and regulation of nitric oxide; CADM1 (rs2027618, p = 3.85E-05) is involved in apoptosis and innate immune response; and PIAS4 (rs199754282, p = 4.57E-05) is important in the Wnt signaling pathway. A supplemental longer list of genetic variants with trending significance is provided (Table S5).

Next, to examine the importance of strictly genome-wide significant genetic variants, we collected a total of 41 distinct genes that were associated with PTSD phenotypes in case-control analysis on European and African ancestry participants, association analysis with total PCL score as the outcome, and meta-analysis of the MVP and Psychiatric Genomics Consortium (PGC) case-control association studies. Among the 41 distinct genes, EFNA5 (rs114851381, p = 1.21E-06), identified in case-control European ancestry of MVP+PGC datasets, seems to be related with impaired angiogenesis and epithelial dysfunction. Further, we explored the 41 PTSD implicated genes (from GWAS-MVP study) with PTSD-correlated modules (identified using the promoter regions of DNA methylation data). Of the 41, 33 genes have one or more promoter CpG probe corresponding to the three modules. One of the modular networks, over-represented by nervous system development among other highly enriched pathways, contains 21 of the 41 genes (21/323 = 6.5%, p = 2.9E-8) (Table S6).

We have also identified SNPs with suggestive significance levels within 1 Mb of each of the significant protein using GWAS summary statistics from PGC for PTSD.^30^ Genome-wide trending associations detected genetic variants at p < 5E-05 located on two significant proteins NRXN3 [rs74503919, chr14] and TGFBR3 [rs201374650, chr1] that were significantly associated with impaired angiogenesis and epithelial dysfunction (Figure 4B). A total of 371 SNPs belonging to other significant proteins have suggestive p values < 5e-03 (Table S7).

### Identification of PTSD-specific genetic controls of protein levels using publicly available protein quantitative trait loci (pQTL) datasets

We have assessed protein expression regulation in the blood of PTSD patients that were dependent on genetic variants. We identified significant and suggestive pQTLs through the analysis of genetic and proteomic data derived from blood samples of PTSD patients and trauma-exposed healthy controls. Genomic region enrichment analysis of the identified pQTL variants revealed 922 minor alleles (loci) that were *cis*-acting pQTLs affecting the expression levels of 46 proteins that have significant difference between PTSD cases and controls. The identified *cis*-acting pQTLs were over-represented among variants suggesting involvement of genetic polymorphisms in regulation of protein expression in PTSD. Significant association of 33 of the 46 proteins in inflammation, oxidative stress, angiogenesis, and cardiovascular functions provides insight into the functional consequences of genetic variation in PTSD.

### Results summary

We evaluated both veterans with or without military-service-related chronic PTSD and pre- and post-deployment active-duty soldiers for molecular and pathway signatures of PTSD. Our approach included integrating longitudinal and cross-sectional multi-omics datasets and selected clinical features of the different cohorts of participants with and without PTSD. The multi-modal molecular signatures and degrees of pathway alterations in blood samples of participants with chronic and more recent onset PTSD were correlated (generally proportionate) to chronicity and symptom severity of PTSD. These findings provided initial evidence that peripheral multi-modal molecular signatures associated with PTSD are indicative of both neuropsychiatric and somatic disorders and are informative of its probable key clinical features and outcomes. Molecular signatures of PTSD cases for males that were overlapping signatures for females were also preserved to some extent across cohorts of veterans and active-duty subjects.

**Figure 5:**
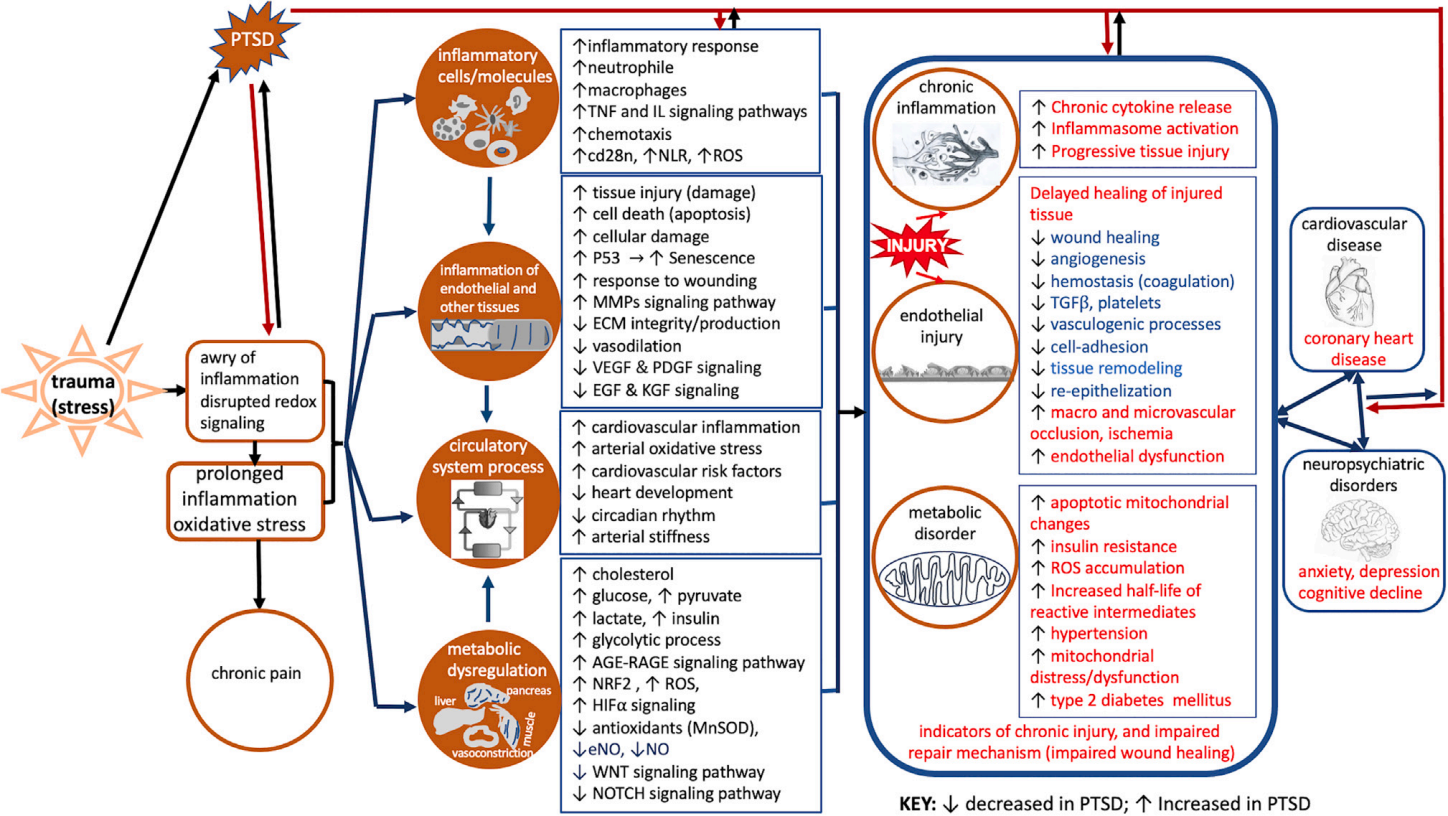
Summary of biological processes and pathways correlated with PTSD clinicals identified in integrated multi-omics analyses across cohorts Multi-omics analyses identified inhibition and activation of specific components of pathways associated with impaired wound healing and comorbidities indicative of chronic inflammation, endothelial injury, and metabolic disorders. Disrupted and prolonged inflammation and redox signaling leading to inflammation and injuries of endothelium and other tissues, metabolic dysregulation, and circulatory system dysfunction. The associated physiological dysregulations correspond with long-term sequelae of PTSD, including cardiovascular disease, T2DM and neuropsychiatric disorders (anxiety, depression, and cognitive decline). Although our study evaluated peripheral markers, the processes identified may either reflect system-wide (including CNS) perturbations or else may lead systemic disruptions related to PTSD pathology. Up arrows indicate upregulation/activation in PTSD; down arrows indicate downregulation/inhibition.

Here, we identified epigenetic patterns (miRNAs and DMRS), proteomic and metabolomic signatures for angiogenesis, response to wounding, inflammatory response, oxidative stress, metabolic dysregulation, and mitochondrial dysfunction that were significantly correlated with severity and chronicity of PTSD symptoms (Figures 3 and 4). The relationship of metabolite and protein signatures and epigenetic scores with PTSD suggest inhibited vasculature morphogenesis and activated inflammation and oxidative stress that are associated with progression and persistence of PTSD.^15^ Inflammation, oxidative stress, and metabolic dysregulation appear to be important signals and mechanisms in causing tissue damage and impaired tissue renewal/repair leading to functional deterioration of vital systems associated with cardiovascular disease, metabolic disorder, and perturbed immune response with disease progression (Figure 5). Replications of these findings in two independent groups of males with different temporal steps of PTSD corroborate the potential roles of identified molecular signatures in mediating the course and persistence of PTSD-related syndromes.

Important pathways such as inflammatory response, oxidative stress, angiogenic signaling, and endothelial dysfunctions also were consistent with postmortem gene expression profiles obtained from discrete brain regions^28^^,^^34^ (Figure 4). Identification of genetic variants belonging to proteins associated with these pathways indicates further that the multi-layer molecular events convergently confirm the differentially altered pathways. These observations corroborate that multi-omics molecular signatures, including proteomic, epigenomic, and metabolomic changes, correlated with PTSD symptoms, have the potential to serve as gradient molecular signatures across temporal and severity trajectories of PTSD.

## Discussion

This study employed an unbiased, integrated, systems-level multi-omics approach to identify molecular signatures with activation levels of enriched pathways in military-service-related PTSD cases compared with controls. Unbiased systems-level molecular approaches hold promise for understanding the molecular underpinnings of PTSD pathogenesis along its clinical courses, severity, and persistence. Altered multi-modal molecular and pathway profiles, identified in the training set of 218 SBC male veterans, were validated in independent case-control comparisons of male and female veterans with chronic PTSD and persisted, though to a lesser extent, in active-duty FCC participants (with recent onset PTSD and subclinical PTSD groups).

Functional analysis of case-control comparisons across cohorts identified pathways implicated in angiogenesis, inflammation, oxidative stress, and metabolism that correlated with PTSD clinical assessments. Inhibition or activation levels of enriched pathways correlated proportionately with clinical indicators of PTSD progression and suggested important molecular profiles corresponding to disease chronicity and severity. Namely, inflammatory responses, oxidative stress, apoptosis, and autophagy were significantly activated across cohorts. Coagulation, platelet activation, and angiogenesis and cell adhesion pathways were inhibited. Several of these enriched pathways and their activation patterns were indicative of impaired wound healing (normal upkeep/repair mechanisms), consistent with an independent meta-analysis study of co-expression networks that identified an aberrant wound-healing module in men exposed to combat trauma.^35^ Metabolic disturbances were reflected in changes in molecular components that are associated with obesity, insulin resistance, T2DM, gluconeogenesis and glycolytic pathways, mitochondrial dysfunction, and decreased cellular energetics (Figure 5). Molecular indicators of inhibited neurogenesis were associated with chronic but not with the more recent nor to the subclinical PTSD (Figure 2E). Some of these pathways—inflammation, oxidative stress, and mitochondrial dysfunction—have been reported in PTSD,^2^^,^^7^^,^^8^^,^^27^^,^^35^^,^^36^^,^^37^^,^^38^^,^^39^^,^^40^ whereas others—endothelial dysfunction, angiogenesis, and wound healing—have been less frequently documented.^35^^,^^41^^,^^42^^,^^43^

These observations raise the possibility that molecular indicators of perturbed angiogenesis (endothelial dysfunction), inflammation, oxidative stress, and mitochondrial dysfunction may play a role in the course and persistence of PTSD including PTSD-related complications later in disease progression (Figure 5). In one hypothetical model, traumatic stress exposure promotes the release of pro-inflammatory cells/molecules leading to an inflammatory response with overproduction of ROS.^15^ Prolonged inflammation and oxidative stress may lead to perturbation in a tetrad of pathophysiological processes, including metabolic dysregulation, endothelial injury (circulatory system damage), inflammation, and oxidative stress.^44^^,^^45^ Over time, the persistence of these molecular changes may lead to long-term sequelae, including cardiovascular disease, T2DM, obesity, insulin resistance, and neuropsychiatric disorders (anxiety, depression, and cognitive decline) (Figure 5).^3^^,^^25^^,^^46^

Overall, this study uses a systems-level, multi-modal approach to describe PTSD course, severity, and persistence as a series of interconnected molecular pathways with discrete protein, metabolite, *cis*-regulatory DMR, genetic, and miR components (Figures 4 and S5B). Reported findings provide support for theoretical models that describe PTSD systemic pathogenesis associated with psychological trauma as an interconnected tetrad of inflammatory responses, oxidative stress, mitochondrial/metabolic dysfunction,^2^^,^^8^^,^^47^ and impaired angiogenesis, which have the potential to lead to cardiovascular sequelae, T2DM, and/or neuropsychiatric disorders over time (Figure 5),^7^^,^^14^^,^^15^^,^^25^^,^^48^^,^^49^ with the caveat that long-term medical outcome data are not available for our subjects. The PTSD tetrad of inflammation, oxidative stress, mitochondrial metabolism, and vascular damage, which underlie impaired wound healing and other comorbidities along disease progression and persistency, are basic features we documented and will continue to describe.

### Molecular indicators of suboptimal vasculature repair

We observed inhibition of angiogenic signaling pathways in PTSD cases (activation *Z* score = −1, q < 7E-11). Impaired angiogenesis and inhibited remodeling of the microvasculature result from changes in the local production of angiogenic/antiangiogenic factors and/or changes in endothelial responsiveness to angiogenic stimuli. PTSD was associated with protein expression signature of impaired angiogenesis in the blood, potentially reflecting microenvironments in the vasculature and organ systems that promote an antiangiogenic phenotype. Decreased expression of VEGF, a major regulator of angiogenesis in many tissues, and/or resistance to the effects of VEGF and platelet-derived growth factor were important indicators for impaired angiogenesis. Decreased expression of NOTCH1 (Figure S4 and Table S3), important in the stabilization of arterial endothelial fate and cardiac valve homeostasis,^45^ further corroborates the negative effect of PTSD on the cardiovascular system. A longitudinal follow-up study of over 7.2 years demonstrated that veterans with PTSD were at increased risk for developing heart failure (hazard ratio = 1.47; 95% confidence interval = 1.13, 1.92) compared with veterans without PTSD after adjusting for age, gender, T2DM, hyperlipidemia, hypertension, BMI, combat service, and military service period.^48^ Decreased angiogenesis is also a risk factor for myocardial infarction and stroke in men and women, independent of depression.^14^

PTSD-associated alterations in endothelial angiogenic phenotypes and circulating biomolecules are also related to mitochondrial dysfunction, factors that may be related to metabolic dysregulation. For example, NADPH oxidases and mitochondria-derived ROS play critical roles in vascular aging by promoting endothelial dysfunction.^50^ Like-wise, the plasma level of the metabolite sphingosine-1-phosphate was differentially expressed in our PTSD cohorts and has network connections with CDH5 (downregulated protein in PTSD cases compared with controls) (Figure 4). Increased levels of sphingosine-1-phosphate may indicate decreased sensitivity of its receptor, sphingosine-1-phosphate receptor 3 (S1PR3). S1PR3 may regulate angiogenesis and vascular endothelial cell function. Previous reports show that elevated expression of S1PR3 in the medial pre-frontal cortex promotes stress resilience by reducing inflammatory processes in rats with chronic social defeat stress. S1PR3 mRNA in blood was reported to be lower in combat-exposed veterans with PTSD, and its expression negatively correlated with symptom severity.^51^

Modulation of these pathways might be effective in improving endothelial function and/or endothelial angiogenic capacity and in restoring potentially impaired processes (in mitigating some aspects of PTSD-related pathophysiological problems). Inflammation, oxidative stress, and metabolic dysregulation also were implicated in tissue injury and dysfunctions in the epithelia of the vasculature system,^15^^,^^52^ potentially contributing to some of the cardiovascular problems^12^^,^^53^^,^^54^ observed in PTSD patients (negatively impacting the health and lifespan of people with the disorder).

### Inflammation and oxidative stress

Molecular signatures of altered inflammation, oxidative stress, integrin signaling, growth factor activity, metabolic dysregulation, and inhibited angiogenesis can potentially affect wound healing and other processes mediating PTSD pathogenesis.^55^ Stress can dysregulate immediate and regenerative immune responses to cause excessive inflammation with functional deterioration of multiple organ systems, including the noninfectious injury to the heart,^13^^,^^56^^,^^57^ arterial inflammation,^58^ and cardiovascular disease.^15^^,^^54^^,^^59^ Inflammatory response after traumatic stress results in the recruitment and activation of leukocytes from the bone marrow and local cytokine production in the vasculature.^13^^,^^15^ Cytokine-mediated neutrophil activation produces ROS, proteases, cytokines, and lipids that propagate endothelial injury. In response, endothelial cells increase expression of adhesion molecules, facilitating leukocyte binding and leading to persistent inflammatory response with impaired wound healing. ROS cause cell death via oxidative stress, and excessive protease activity leads to increased degradation of the extracellular matrix. Our study reports an increase in apoptotic activity and increased expression of matrix metalloproteinase 9, an important mediator of remodeling after endothelial injury.^60^ Aberrant cellular apoptosis and matrix degradation compromise endothelial structural integrity and result in a maladaptive response to wounding. The innate immune response can be further activated in response to apoptosis and matrix degradation products leading to chronic inflammatory response.

In addition to the systemic consequences of activated inflammation as a whole, individual cytokines were reported as important signatures for combat-related PTSD onset and risk. For example, significantly altered pro-inflammatory chemokines have been implicated as markers of PTSD onset, risk, and resilience in the US military service members deployed to Iraq and Afghanistan.^61^ CCL2, which was increased in SBC and decreased in FCC, and CCL15 and CXCL12, which were decreased in SBC and increased in FCC, were associated with PTSD in the first year following deployment. CCL25, which was increased in both SBC and FCC cohorts, was positively correlated with PCL scores.^61^ Other classes of pro-inflammatory -molecules, including complements and complement receptors including C2, C3, and C5, which have been reported in neurodegenerative disorders and delaying wound healing,^62^ showed modest but consistent increase in the cases of the SBC and FCC cohorts.

We also observed increased activation of oxidative stress (*Z* score = 1.93, q < 6.4E-3)^63^ and decreased expression of ROS-scavenging proteins including SOD2. NADPH oxidases are major sources of ROS implicated in T2DM, hypertension, hypercholesterolemia, and aging. High NADPH oxidase activity in leukocytes may contribute to increased systemic inflammation and early vascular aging.^44^ Impaired antioxidant activity was indicated by decreased SOD2 expression, lower circulating L-arginine,^50^ and increased phosphorylation of ERK1/2.^44^ Mitochondria-derived oxidants can lead to oxidative stress as well, independently of NADPH oxidase, and result in increased phosphorylation of ERK1/2, increased MAPK activation, and production of inflammatory cytokines, contributing to suboptimal mitochondrial activity and energy metabolism.^64^

### Metabolism and energy homeostasis

We observed increased levels of insulin and glucose in participants with PTSD (Figure 4 and Tables S2 and S3). Hyperglycemic conditions, along with reduced expression of insulin receptors, are markers of decreased insulin signaling. As a precursor to T2DM, insulin resistance may be indicative of glucolipotoxicity associated with β cell dysfunction.^18^ Under insulin-resistant states, insulin response is impaired in liver, skeletal muscle, white adipose tissue, the vasculature, and the kidney leading to hyperglycemia, hyperinsulinemia, high plasma free fatty acid levels, and inflammation-activated serine/threonine kinases. Either directly or via lipid intermediates, these adaptor proteins and phosphatases lead to mitochondrial dysfunction or the induction of endoplasmic reticulum stress (Tables S2 and S3). Activation of these negative regulators results in chronically reduced cellular response to insulin.^18^

Other pathways related to insulin signaling such as PI3K-AKT, MAPK, PKC, ERK1/2, and gluconeogenesis were also altered in our PTSD cohorts. In the setting of insulin resistance, various metabolic and inflammatory factors inhibit the actions of insulin receptor targets, leading to reduced activation of the PI3K-AKT axis. The resulting reduction in nitric oxide (NO) production leads to impaired vasodilation, further shifting the insulin signal from the PI3K axis to the MAPK axis. Activation of PKC in insulin-resistant individuals results in phosphorylated endothelial NO synthase (eNOS), which negatively regulates further NO production. The MAPK axis alters expression of the vascular cell adhesion molecule. Dysregulation of insulin-regulated pathways culminates in and sustains the pathophysiological alterations found in metabolic syndrome, such as reduced endothelial function, mitochondrial dysfunction, and inhibited glycolytic processes, which are changes observed early in the course of the development of T2DM.^18^

Insulin-resistant states, as a feature of metabolic dysregulation, can exacerbate elevated inflammation, oxidative stress, and impairments in angiogenesis and wound healing processes potentially contributing to persistent PTSD and related medical comorbidities. Under glucolipotoxicity conditions, cell proliferation and migration are decreased, ROS production is increased, AKT phosphorylation is decreased, and ERK1/2 phosphorylation is increased, leading to impaired fibroblast proliferation and migration mediated by oxidative stress and hence to delayed wound healing in diabetic conditions.^15^^,^^46^ These results provide support for the hypothesis that the PTSD tetrad of symptoms are inter-related at the molecular level.

We also observed increased plasma lactate and pyruvate and decreased citrate levels, suggesting impaired mitochondrial Krebs cycle/TCA cycle, which may also indicate a pro-diabetic state or obesity or shunting of pyruvate metabolism from aerobic (high ATP production) to anaerobic (low ATP production) metabolism.^3^^,^^7^^,^^65^ Hyperglycemia and chronic inflammation fuels ERK1/2 signaling coupled with glycolysis in pro-inflammatory macrophages, which contribute to the expansion of white adipose tissue leading to insufficient vascular remodeling. Hypoxia (one of the significant pathways; Figure 2G) increases lactic acid production by anaerobic metabolism, which was shown to be higher in obese mice than in lean mice. Generally, adipose hypertrophy, hyperglycemia, and chronic inflammation exacerbate -insulin resistance and metabolic disorders.^66^

Additionally, we observed decreased levels of polyunsaturated fatty acids, omega-3 fatty acids, and essential fatty acids in PTSD cases compared with controls (Figure 4). Unsaturated fatty acids are implicated in both the wound healing process and insulin resistance states. Previous direct evidence has shown that fatty-acid-induced gut-brain signaling attenuates neural and behavioral effects of sad emotion in humans.^67^ Fatty acids regulate lipid metabolism and cellular differentiation and proliferation, in addition to contributing to metabolic syndrome-related disorders (e.g., insulin resistance and hypercholesterolemia).^67^ Our multi-modal integrative analyses identified that proteins associated with the fatty acid metabolites (Figures S5B and 4) may also play a role in PTSD-related somatic pathologies. Collectively, the reported multi-omics integrative profiling is consistent with metabolic dysregulation along with reduced energy production in PTSD cases compared with controls.

### Potential genetic risk factors associated with significant proteins (altered pathways)

Potential genetic risk factors were found to be related with proteins significantly associated with wound healing and inflammatory response (PIK3CG and GRB2), neurogenesis (CAMK1D and NCAM1), cognitive disorders (KIRREL3, CADM1, ROBO2, and C3), vasculature development/angiogenesis (HS6ST1 and EFNA5), oxidative stress, and regulation of nitric oxide (MB). These associations are important indicators of the potential roles of significant genetic variants in regulating the corresponding proteins and their probable roles as risk factors. For example, genetic variations at the PIK3CG loci have been associated with attention-deficit/hyperactivity disorder,^68^ CAMK1D loci in T2DM,^69^ NCAM1 loci in cardiovascular problem,^70^ NRXN3 loci in neurodevelopmental/neuropsychiatric disorders,^71^^,^^72^^,^^73^^,^^74^ cardiovascular disease,^75^^,^^76^^,^^77^^,^^78^ and variants at TGFBR3 loci are shown to be risk factors in schizophrenia, bipolar, and major depressive disorder,^79^ vasculature development, and cardiovascular health.^80^^,^^81^ These evidences support that genetic variants associated with significant proteins might underlie PTSD predisposition.

Taken together, the combined processes associated with angiogenesis, inflammation, oxidative stress, endothelial dysfunction, metabolic dysregulation, and other somatic/physiological pathologies in the acute presentation of PTSD may lead to cardiovascular and neuropsychiatric complications in the later progression of the disorder. The data in the recent onset PTSD cohort allowed us to identify molecular indicators that were consistent with the literature, including pathways indicative of tissue injury and suboptimal vasculature repair. Inflammation and ROS production may contribute to tissue damage, including endothelial cell dysfunction and vasculature damage, which may progress to cardiovascular morbidities and impaired neurogenesis. The integrated analysis facilitated discovery and description of the interconnections among pathways significantly correlated with facets of PTSD-related symptoms. We speculate that vasculature damage and related physiological pathologies potentially due to inflammation and oxidative stress may precede neurological sequelae (e.g., cognitive decline). Our findings raise the possibility that tissue injury and aborted healing of the injured tissue may contribute to the onset, course, and persistence of PTSD including important comorbidities (cardiovascular diseases, metabolic dysregulation, T2DM, chronic pain, and neuropsychiatric disorders) (Figure 5).

Although PTSD has primarily been conceptualized as a brain disease, it is increasingly being recognized as a systemic condition affecting multiple physiological parts and associated with divergent chronic medical conditions.^7^^,^^82^^,^^83^^,^^84^ The present findings are consistent with systemic physiological perturbations in PTSD. The relevance of these peripheral markers to brain biochemistry and function is uncertain. However, numerous lines of evidence suggest that even peripheral oxidative, inflammatory, vascular, and metabolomic dysregulation can affect brain function and perpetuate PTSD symptoms.^37^^,^^38^^,^^39^^,^^85^^,^^86^^,^^87^^,^^88^ Thus, PTSD is coming to be seen as a systemic disorder rather than as a purely psychological illness.^7^^,^^8^^,^^89^

### Conclusions

We identified reproducible molecular signatures of combat-related PTSD including sequence-specific genetic variants, epigenetic marks, microRNA, and proteomic and metabolomic features indicative of inhibited angiogenesis, activated inflammation, oxidative stress, and metabolic dysregulation. Degrees of alterations of these multi-modal molecular signatures in blood samples of participants with chronic and more recent onset PTSD were significantly correlated with the different spectra of disease progression and were generally proportionate to severity and chronicity of the different facets of PTSD symptoms.

The altered molecules and pathways indicated interconnection and convergence of the underlying molecular mechanism and provided relations between psychiatric and somatic comorbidities (associated with combat-related PTSD) at the molecular level. Namely, molecular signatures of elevated inflammation and oxidative stress, metabolic dysfunction, and inhibited angiogenesis were implicated in tissue injury including impaired tissue repair and epithelial dysfunctions of the vasculature system. Tissue injury and impaired healing of the injured tissue may contribute to the onset, course, and persistence of PTSD including comorbidities: cardiovascular diseases, T2DM, chronic pain, and neuropsychiatric problems. These observations support that multi-omics features that were significantly altered and correlated with PTSD symptoms seem to mediate the course and persistence of PTSD in addition to being potential markers across its temporal and severity trajectories. Such a systems-level understanding of PTSD can help to address PTSD-associated dysfunctions in a concerted approach and may contribute to developing prevention, diagnosis, and treatment strategies.

There are multiple strengths of this study, including (1) the use of well-characterized cohorts of military personnel that cover a spectrum of severity and chronicity of PTSD symptoms. (2) There was systems-level integration, based on multi-omics profiling, to interrogate circulating molecular signatures of PTSD, including both regulatory elements (DMRs and miRs) and functional components (metabolites and proteins). Importantly, the molecular signatures -correlate with the clinical diagnostic criteria for PTSD. (3) Data from females in both active-duty and veteran cohorts were available for qualitative comparisons with the male military training, validating, and testing cohorts. (4) This study was conducted in a group where some of the socio-economic factors such as diet, poverty, and systemic racism were less likely to confound the findings.

### Limitations of the study

Limitations of the study include (1) the smaller sample size of female cohorts and sex-related unmeasured potential confounders such as menstrual cycle or hormonal drugs. (2) This study was focused on veterans and active-duty service members, and it is not clear if the findings will generalize in the wider civilian population. Particularly, they may not generalize well for populations most at risk, which also have significant exposures to other environmental and psychosocial factors that contribute to adverse stress-related behavioral and physical health outcomes. (3) The non-PTSD control groups had all been exposed to PTSD diagnostic criterion A-level combat trauma, yet they had not developed PTSD. Therefore, they may represent an atypical highly resilient group, as opposed to a non-exposed healthy control group (except the longitudinal sub-cohort). Having a non-exposed control group would be important in deducing whether the molecular signatures we observed relate to PTSD in the cases or, rather, to resiliency in the controls. In any event, having combat-trauma-exposed controls facilitates interpretation of our data, as pointing to the development of PTSD, rather than non-specific effects of trauma exposure itself, in producing the observed effects. (4) Although several of the physiological perturbations we observed may predispose to the development of serious medical conditions such as diabetes mellitus type II (DMII), cardiovascular disease (CVD), immune dysfunction, etc., we do not have long-term medical follow-up data on participants to determine if these perturbations did in fact presage such conditions in our participants. The longitudinal sub-cohorts have smaller n and were followed for a relatively short period (<2 years). (5) Besides the first three principal components from GWAS ancestry genotype, datasets were also corrected for the confounding effect of “self-reported race,” a social construct, and may not reflect the actual ancestry.

## Consortia

PTSD Systems Biology Consortium: Victor I. Reus, Mazen Istanbouli, Allison Hoke, Stacy Miller, Linda Petzold, Guia Guffanti, Taek Kyun Kim, Kelsey Dean, Linda Bierer, Nabarun Chakraborty, Gwyneth Wu, SysBioCube.

## STAR★Methods

### Key resources table

**Table:** 

REAGENT or RESOURCE	SOURCE	IDENTIFIER
**Biological samples**		

PTSD Biomarker Consortium Samples from Case-control cohorts	Dean et al.^27^	https://doi.org/10.1038/s41380-019-0496-z
New York University Samples from active duty soldiers	Schultebraucks et al.^31^	https://doi.org/10.1038/s41380-020-0789-2

**Chemicals, peptides, and recombinant proteins**		

1N Sodium Hydroxide Solution	Fisher Scientific, USA	SS277

**Critical commercial assays**		

SomaLogic 1.3K proteins	SomaLogic®, Inc., Boulder, CO	Home - SomaLogic
Qubit dsDNA BR (Broad Range) Assay Kits	Thermo Fisher, USA	Cat# Q32853
EZ96 DNA methylation kit	Zymo Research, Orange, CA, USA	Cat #D5004; https://www.zymoresearch.com/
HumanMethylation450 BeadChip	Illumina Inc., USA	https://www.illumina.com/content/dam/illumina-marketing/documents/products/datasheets/datasheet_humanmethylation450.pdf
Zymo Genomic DNA Clean & Concentrator- 5 kit	Zymo Research, Orange, CA, USA	Cat #D4067; https://www.zymoresearch.com/
QIAamp DNA Blood midi kit	Qiagen, USA	Cat # 51185; https://www.qiagen.com/us/products/

**Deposited data**		

Million Veteran Program (MVP) - PTSD GWAS summary statistics consists of 186,689 participants for quantitative analysis and 214,408 (algorithmically defined 36,301 cases and 178,107 controls) total participants	Stein et al.^29^	N/A
Psychiatric Genomic Consortium (PGC) - PTSD GWAS summary statistics freeze-2 summary statistics data for European-ancestry participants (23,212 cases and 151,447 controls)	Nievergelt et al.^30^	https://pgc-ptsd.com/
Psychiatric Genomic Consortium (PGC) - MDD GWAS summary statistics data (59,851 cases and 113,154 controls)	Wray et al.^90^	https://pgc.unc.edu/for-researchers/working-groups/mdd/
UK Bio Bank broad GWAS depression summary statistics data (113,769 cases and 208,811 controls)	Howard et al.^91^	http://www.ukbiobank.ac.uk/
postmortem gene expression data from brain-subregions of PTSD cases and controls	Girgenti et al.^28^	https://www.nature.com/articles/s41593-020-00748-7
Multi-omics and clinical datasets generated from this study	SysBioCube	https://sysbiocube-abcc.ncifcrf.gov

**Software and algorithms**		

R programming versions 4.1 & 4.2	The R Project for Statistical Computing	https://www.r-project.org/
Manuscript custom R code	Github	https://github.com/smuhie/multi-omics/blob/main/hPTSD_Transcriptome.R
ggplot2,^92^ weighted gene correlation network analysis (WGCNA),^93^^,^^94^ ComplexHeatmap,^95^ circlize^96^ igraph^97^ metaboanalystR,^98^^,^^99^ biomaRt,^100^ edgeR, Limma,^10^ ChAMP v2.14.0, RnBeads, MatrixEQTL,^101^ minfi v1.30.0	Comprehensive R Archive Network & Bioconductor v3.16	https://www.r-project.org/; www.bioconductor.org
KEGGscape, Bingo (GO) and Reactome FI	Cytoscape version 3.9 packages	www.cytoscape.org
Python versions 3.9 & 3.11	Python Software Foundation	https://www.python.org
Gephi^92^ version 0.10.1	The Open Graph Viz Platform	https://gephi.org/
SPSS	IBM, Armonk, NY	https://www.ibm.com/spss/
Ingenuity Pathway Analyses (IPA)	QIAGEN Redwood City, CA	https://digitalinsights.qiagen.com/products/qiagen-ipa
NetworkAnalyst	Open-source online software	https://www.networkanalyst.ca

### Resource availability

#### Lead contact

Further information and requests for resources and reagents should be directed to and will be fulfilled by the lead contact, Dr. Marti Jett (marti.jett-tilton.civ@health.mil).

#### Materials availability

This study did not generate new unique reagents.

### Experimental model and subject details

#### Subjects and informed consent of participants

Case-control cohorts were recruited as part of the PTSD Biomarker Consortium as previously described.^2^^,^^27^^,^^31^ For all cohorts, study procedures were approved by the Institutional Review Board of NYU Grossman School of Medicine, as well as the Human Research Protection Office of the United States Army at Fort Detrick, Maryland and Army Command of the 101st Airborne at Fort Campbell, Kentucky. Participants were given written informed consent. All works and consents were obtained with the approval of the involved Institutional Review Boards. Ethical principles for the conduct of human research were followed as described.^31^ And the investigators have adhered to the policies for protection of human subjects as prescribed in AR 70–25.

#### Cohort description

Veterans were recruited from Operation Enduring Freedom (OEF) and/or Operation Iraqi Freedom (OIF). Study approval, recruitment processes, inclusion criteria, clinical data collection, and clinical assessment parameters for veteran participants have been previously described.^2^^,^^27^ Briefly, participants met the Diagnostic and Statistical Manual of Mental Disorders DSM-4 PTSD criteria for current warzone-related PTSD for at least 3 months duration, and a Clinician-Administered PTSD Scale (CAPS) total score ≥40. PTSD-negative controls were combat-exposed (OEF/OIF) veterans who were negative for lifetime combat or civilian PTSD and had a current CAPS total score <20.

Active-duty soldiers (n = 180) were recruited from the 101st Airborne at Fort Campbell, Kentucky and were assessed before and after being deployed to Afghanistan in February 2014. This longitudinal study followed-up recruited participants at three different phases (temporal steps). The first phase of recruitment occurred during a 2-week period immediately prior to deployment in February 2014. The second phase occurred 3 days after returning from a 10-month tour of duty. The third phase occurred 90 to 180 days post-deployment. The deployment history and inclusion/exclusion criteria of participants are detailed earlier.^31^ PTSD symptoms were assessed using the validated PTSD Checklist (PCL), a 17-item, DSM-5-based self-report measure. The rapid tempo of the deployment and limited time with each participant precluded administering a structured diagnostic interview. Current probable PTSD diagnosis was based on a PCL total score ≥38. Participants with PCL ≥22 and PCL <38 were regarded as subthreshold PTSD, whereas subjects with PCL <22 were controls. Cut scores were based on a conservative application of recommendations for screening for PTSD with the PTSD CheckList for DSM-5 (PCL-5) in active-duty military personnel (Wortmann, Psychological Assessment, 2016), Demographic data, such as age, gender, and self-reported race, were also collected. Controls (non-PTSD) were combat exposed and age-, self-reported race-, deployment time-, and sex-matched participants.

#### Cohort composition

The chronic PTSD cohorts included veteran participants recruited into 3 independent case-control cohorts (Figure 1). The Systems Biology Cohort (SBC) Training (n = 218) included combat-exposed male OEF/OIF veterans with PTSD (n = 109) and age-matched combat-exposed male OEF/OIF veterans without PTSD (n = 109). The SBC Testing (n = 82) included combat-exposed male OEF/OIF veterans with PTSD (n = 43) and age-matched, combat-exposed male OEF/OIF veterans without PTSD (n = 39). The participants in the training and testing sets of SBC were recruited and samples were collected independently two years apart. The SBC Female (n = 40) included combat-exposed female OEF/OIF veterans with PTSD (n = 19) and age-matched, combat-exposed female OEF/OIF veterans without PTSD (n = 21).

Recent PTSD, Fort Campbell Cohort (FCC), included active-duty participants divided into 4 case-control groups (Figure 1). The FCC Validation (n = 91) included combat-exposed male Fort Campbell active-duty participants with PTSD (n = 47) at 3-day or 90 to 180 days post-deployment (most post-deployment samples were phase 3, 90–180 days) and male Fort Campbell active-duty participants without PTSD (n = 44) 2 weeks pre-deployment. The FCC Subthreshold (n = 112) included combat-exposed male Fort Campbell active-duty personnel with subthreshold PTSD (n = 68) and male Fort Campbell active-duty personnel without PTSD (n = 44) 2 weeks pre-deployment. The n = 44 participants without PTSD in the FCC Subthreshold group were the same n = 44 participants included in the matched control group in the FCC Validation group. The FCC Longitudinal (n = 26) were Fort Campbell male active-duty personnel cohorts with PTSD after combat exposure at 90 to 180 days post-deployment (case; n = 26 phase 3 samples) and the same participants before combat exposure at 2 weeks pre-deployment without clinical PTSD (control; n = 26 phase 1 samples). The FCC Female group consisted of combat-exposed female Fort Campbell active-duty participants with PTSD (n = 8) and female Fort Campbell active-duty participants without PTSD (n = 13) 2 weeks pre-deployment. Demographic composition of both SBC and FCC cohorts given (Table S4).

#### Inclusion of civilian cohort for qualitative comparison

The Civilian cohort included 19 male participants: 10 PTSD+ (CAPS D Combined Life current ≥ 15), and 9 PTSD- (CAPS D Combined Life current ≤ 5). These participants were recruited as part of the Grady Trauma Project and included for qualitative comparison with the veteran and active-duty cohorts. Since the n of civilian is small, we excluded them from the main text.

The civilian participants were few in number and seem to be more heterogeneous (as it was inferred from their molecular datasets). And yet we observed consistency with other cohorts in terms of enrichment significances of some of the core pathways (Figure S5). But not so consistent in terms of activation directions. It seems that much larger N needed to identify reliable PTSD signal to overcome the diverse trauma types and heterogeneity of civilian participants.

Overall, the sampling strategies of this study were designed to include participants with chronic PTSD and those with more recent trauma exposure, including those with a spectrum of symptom severity, providing an opportunity for identifying molecular signatures and pathways that were altered across the course of disease progression.

### Method details

Cohorts, clinical assessment, and blood collection/processing assays and data analysis steps are shown diagrammatically in Figure 1.

#### Clinical assessments

Active-duty cohorts were clinically assessed prior to stressor exposure during the index deployment as well as at post-stress exposure phases, and included sex, age, self-reported race, education, and BMI, as well as clinical self-report.^31^ In addition, participants were evaluated for psychological symptoms and functioning, attention, emotion regulation, and executive function. Comprehensive whole blood, plasma, serum, and buffy-coat markers were collected. Complete blood count, lipid panel, inflammatory markers, liver functioning tests, metabolomics, and methylation markers were assessed.^31^

#### Blood draws (from both veterans and active-duty)

Blood samples were drawn in the morning after a night of fasting in appropriate collection tubes, and were processed and aliquoted for storage into whole blood, serum, plasma, buffy coat or peripheral blood mononuclear cells (PBMCs) depending on the assay (Table 1 and S8). Blood samples for serum separation were collected in SST tubes and were processed following the manufacturer’s protocol. EDTA plasma was used for metabolomics assays. PAXgene DNA tubes were collected for DNA isolation. Samples were inventoried and stored frozen at −80°.

#### Molecular assays and data analysis

Blood samples were assayed for proteomics, DNA methylomes, metabolomics, microRNAs, immune cell counts, endocrine markers, cytokines, and routine clinical labs. Data were normalized and cleansed; covariate analysis was conducted; and weighted gene correlation network analysis (WGNCA) was conducted to identify modular networks and module preservation across cohorts. Unbiased pathway enrichments, activation status of significant pathways and correlations of pathways with PTSD clinicals were determined.

#### Serum samples processing for SomaLogic proteomic assays

Whole blood samples were drawn directly into SST tubes via standard phlebotomy technique and all serum samples from SST tubes were processed following standard serum isolation procedures. Briefly, this procedure involved inverting SST tubes 5 times and leaving them at room temperature for 30 min to allow clotting, followed by centrifuging for 10 min at 1300rcf in a swinging bucket rotator at room temperature. Isolated serum samples were aliquoted and immediately stored at −80°C until use. Other data types, such as routine clinical lab values and physiological measurements, were collected using standard procedures.

### Quantification and statistical analysis

#### Proteomic assays

Proteins were evaluated using Aptamer based assay using SomaLogic platform consisting of 1.3K proteins (SomaLogic, Inc., Boulder, CO). Serum samples isolated from whole blood, were randomized across 7 high throughput 96 well plates (to minimize batch difference). Sample aliquots (160ul) were shipped frozen to the Center for Human Immunology and Autoimmunity, and Inflammation (CHI) facility, National Institute of Allergy and Infectious Disease, NIH (Bethesda, MD, USA) for the SOMAscan proteomic assay. The process for proteins assay and raw data normalization has been described.^102^^,^^103^ Each 96 well plate had CHI quality control (QC), SomaLogic calibrators, SOMAscan QC and no protein/buffer only controls. Quality control and calibrators are pooled samples composed of the same matrix as the biological samples being measured in the plate. Expression levels of 1305/1322 proteins/probes from cryopreserved serum samples were assayed on a 1.3K SOMAscan hybridization microarray platform (SomaLogic, Inc., Boulder, CO). The proteomics data were expressed as abundance in relative fluorescence units.

#### Normalization and data cleansing

Proteomics data were normalized in 4 steps using 1) hybridization normalization which removes variability in the readout of individual microarrays; 2) median signal normalization which removes inter-sample differences within a plate due to technical differences such as pipetting variation and 3) between-run calibration normalization which removes variance across assay runs; and 4) inter-plate normalization using CHI QC which is performed to allow normalization across experiments.^102^^,^^103^

#### Covariate analysis for confounders and filtering of differentially altered proteins between cases and controls

Covariate analysis was used to identify potential confounders. We applied regression methods for assessing covariates between PTSD cases and controls for BMI, age, ancestry, self-reported race, smoking/cotinine, mild TBI, BDI total, education, and sample collection/processing batches. Network modules identification and cutoffs for significances were done at the protein and pathway levels. The less significant protein from modules were filtered out by intersecting with a list of significant proteins obtained through comparative analysis of PTSD cases vs. control in each cohort. Overall, multi-layer stringency was used for inclusion of protein nodes and corresponding pathways.

#### Weighted gene correlation network analysis (WGCNA) for identification of modular protein networks and module preservation across cohorts

Weighted gene co-expression network analysis (WGCNA) was used to identify modular networks of proteins based on a correlation of protein expression profiles.^104^ Networks were created using the WGCNA R package’s block-wise Modules function with a soft-thresholding power of 4 to create unsigned networks with a minimum module size of 30 in the SBC Training cohort. The modules, in each network, were evaluated for preservation in the other cohorts using the WGCNA R package’s module preservation function with 300 permutations and using each of the originally identified networks as the reference network. Modules were visualized by selecting the top significant proteins and nodes with the highest soft connectivity within the module and filtering out any connections between those nodes with a correlation <0.05. Soft connectivity was calculated as the sum of correlations between the expression of a protein and all other proteins in the module. The detailed step-by-step procedures of WGCNA analyses and module preservation calculations are given.^93^^,^^94^

#### Comparability of protein datasets across cohorts

Protein datasets from different cohorts were assessed by correlating measures of average protein expressions and overall connectivity as indicators of similarities and to ascertain comparability.^94^ Modular networks were identified using datasets from the SBC Training cohort (veteran cohorts of N = 218: 109 cases and 109 controls) (Figure S1).

#### Choosing the soft-thresholding power: Analysis of network topology

Undirected correlation was used to identify co-expressed networks (modular protein sets). Co-expression similarity was raised to the soft thresholding power of 4 to calculate adjacency to identify weighted protein networks. The soft thresholding power 4 was chosen based on the criterion of approximate scale-free topology (using the function pick SoftThreshold that performs the analysis of network topology which aids in choosing a proper soft-thresholding power) (Figure S2).

#### Calculation of Topological Overlap Matrix (TOM)

To minimize the effects of noise and spurious associations, we transformed the adjacency in Topological Overlap Matrix, and calculated the corresponding dissimilarity. Then hierarchical clustering was used to produce a hierarchical clustering tree (dendrogram). Branches of the dendrogram group densely interconnected, highly co-expressed proteins together. Module identification amounts to the identification of individual branches (“cutting the branches of the dendrogram”) using the Dynamic Tree Cut for branch cutting from the package DynamicTreeCut. Similar modules that were highly co-expressed and highly correlated based on their eigengenes were merged.

#### Calculation of consensus topological overlaps across datasets

The consensus Topological Overlap across datasets was calculated by taking the component-wise (“parallel”) minimum of the TOMs in individual datasets. Thus, the consensus topological overlap of 2 proteins is only large if the corresponding entries in the other datasets are also large.

#### Qualitative and quantitative measuring of network preservation at the module level

Using WGCNA variables from the SBC Training dataset and module definitions, we assessed how well modular networks identified in the SBC Training dataset were preserved in datasets across the other 3 cohorts (SBC Testing, FCC Validation and FCC Subthreshold). As a qualitative assessment, we imposed the modules from SBC Training on the network for the dataset from the other cohorts, and then plotted the resulting networks. These module labels still grouped together in datasets of other cohorts and were highly preserved. To quantify module preservation, we took advantage of the WGCNA built-in module Preservation function to assess how well a module in SBC Training was preserved in the other studies by calculating *Z* score summary. We assessed the preservation of each module in each of the other cohorts (SBC Testing, FCC Validation, and FCC Subthreshold) using module preservation *Z* score.^105^ For example, the preservation of each module from SBC Training cohort was calculated in the SBC Testing, FCC Validation and FCC Subthreshold cohort’s datasets, thus creating a comprehensive quantitative measure of similarity between every module of every network (Figures 2A, 2B, and S3B). A module was defined as highly preserved in another cohort if its preservation *Z* score was above 10, and moderately preserved between 5 and 10.^105^

#### Intramodular analysis: Identifying proteins with high protein significance and module membership

Using the protein significance and module membership measures, we identified proteins that were significantly altered in PTSD cases as well as module membership in the modules significantly correlated with PTSD clinical variables. We plotted scatterplot of protein significance versus module membership in the 4 identified modules that were highly correlated with PTSD (Figures 2A, 2B, and S3B). Modules with high association with PTSD symptoms and clinical variables were identified, and their central players by the module membership measure.

Persistency (across-cohorts) of modular networks and member proteins of modules that were significantly correlated with PTSD clinicals were checked using protein expression parameters (including assessing their significance levels and direction of expression or fold changes).

#### Relating modules to external clinical traits

##### Quantifying module – PTSD associations

To identify protein modules that are significantly associated with PTSD, the summary profile (eigengene)^106^ for each module was correlated with clinical variables looking for the most significant associations. We quantified associations of modular networks with PTSD by defining member proteins significance as (the absolute value of) the correlation between the module and the clinical measurements relevant to PTSD. For each module, we also defined a quantitative measure of module membership as the correlation of the module eigengene and the protein expression profile. This allowed us to quantify the similarity of all proteins on the platform (proteins that passed normalization QC) to every module.

#### Using functional and trend associations

Functional grouping of proteins met 2 criteria: proteins with the same functions as the significant proteins (as evidenced in the literature by direct experiments), and which also have the same directional expression (trending in the same direction as their co-functional significant proteins).

#### Meta-analyses across cohorts on multiple conditions

Two meta-analysis methods were used to identify proteins persistent, mainly, across SBC Training, SBC Testing and FCC Validation cohorts.

##### Combining p Values

Stouffer’s combined probability test followed by Benjamini-Yekutieli’s correction for multiple hypothesis testing was used to identify proteins that had a q-value <0.05 across SBC Training, SBC Testing and FCC Validation cohorts. Stouffer’s method (based on inverse normal transformation) incorporates weight (i.e., taking into account the sample size of each cohort) into the calculation, which usually gives more sensitive and better results.

##### Combining effect sizes

Random effects model (REM), which gives more conservative results with more confidence, was used. REM’s effect size is based on the difference between 2 group means divided by standard deviation. The estimated effect size in each cohort was assumed to come from an underlying true effect size plus measurement error, in addition to the assumption that each cohort contains a random effect that can incorporate unknown cross-study heterogeneities in the model (which could be due to a batch or cohort difference). Statistical heterogeneity, in this model, were estimated using Cochran’s Q tests.

#### Filtering for proteins with the same expression directions across cohorts

Proteins with the same expression directions across cohorts (that were also at least somehow significant p < 0.1) were selected as an additional filtering approach.

Summary of Methods used for filtering proteins used for down-stream pathway and correlation analyses.

#### Identification of differentially expressed proteins that were persistent across cohorts

We employed 3 major steps and approaches to identify significant proteins that were persistent across (at least the three main cohorts of participants: SBC Training, SBC Testing, and FCC Validation).

1. Identification of modular networks in the training cohort that were preserved in the SBC Testing, FCC Validation and FCC Subthreshold cohorts (Figure S3). Relevant modular networks were identified by correlating each of the preserved module with PTSD and other important clinical measurements. Four of the six preserved modular networks were significantly correlated with PTSD, and clinical symptoms: re-experiencing, avoidance and hyperarousal. Then significant member proteins were identified by intersecting member proteins of each of the four (PTSD relevant) modules with list of proteins that were filtered using FDR correction at q < 0.1 in the SBC Training cohort. These sets of proteins were found to have the most overlap with the list of proteins identified using independent meta-analyses algorithms (described next) across the SBC Training, SBC Testing, and FCC Validation cohorts.
2. We carried out independent meta-analyses across the SBC Training, SBC Testing and FCC Validation cohorts using two main algorithms: (i) Stouffer’s method of combining *p* values (based on inverse normal transformation which incorporates weight based on sample size of a cohort) which is more sensitive that Fisher’s; (ii) combining effects sizes, using random effects models which was selected based on statistical heterogeneity estimated using Cochran’s Q tests.
3. Using functional and trend associations. Functional grouping of proteins which met two criteria: proteins with the same functions as the significant proteins (as evidenced in the literature by direct experiments), and at the same time they have the same directional expression (trending in the same direction as their co-functional significant proteins).

#### Dimension reduction to find correlation scores for each pathway (using WGCNA function)

Correlation values for each pathway was obtained by reducing pathway data matrix to vectors – that is, if there are 40 proteins associated with inflammation (a mix of up and down regulated proteins corresponding to a specific subject or sample), then finding the resultant value of all 40 proteins for that specific subject results a single value (here, we are more interested in the resultant effect of a specific pathway on each of the subjects/samples) – so inflammatory pathway will have a single value for each subject or sample (a vector of values corresponding to subjects); the idea of converting matrix to vector follows the principle of dimension reduction without loosing much of the important information (which can be done using a linear algebra principle of calculating eigenvalue given a constraint condition).

If there is a matrix A and vector u, then A u = λu whereas λ is the eigenvalue

which means for non-zero u, A can be represented by λ for a given value of u to make it clear using very simple example of a 2 × 2 matrix; A = $\left[\begin{array}{c} 2\;1\\ 4\;2 \end{array}\right]$ andu = $\left[\begin{array}{c} 1\\ 2 \end{array}\right]$ then A u = $\left[\begin{array}{c} 4\\ 8 \end{array}\right]$ = 4 $\left[\begin{array}{c} 1\\ 2 \end{array}\right]$ which means λu = 4 $\left[\begin{array}{c} 1\\ 2 \end{array}\right]$

Hence λ = 4 (eigenvalue) given that u = $\left[\begin{array}{c} 1\\ 2 \end{array}\right]$ which is the eigenvector.

That is, the matrix A has an eigenvalue of 4 for the eigen vector, u = $\left[\begin{array}{c} 1\\ 2 \end{array}\right]$, and the A $u=\left[\begin{array}{c} 4\\ 8 \end{array}\right]\text{,}$ is a column vector, which is the product of the 2 × 2 matrix with the eigenvector, belonging to the eigenvalue 4. For different values of u,thevalueofλ change (value of λ for A is constrained by the values of theu vector).

Integration of significant proteins with other omics (microRNA, DNA methylation, metabolomics) datasets.

##### Identification of differentially expressed miRs and regulatory pairing with significant proteins

MiR datasets were analyzed for significance using edgeR (R package) or moderated t-test by adjusting for ancestry and BMI using the Limma (R package) comparing combat-exposed PTSD-positive to combat-exposed control for the PTSD effect. The search for down-stream regulatory targets for differentially expressed miRs among significant proteins was done by connecting to the databases: TargetScan Human, TarBase, miRecords and Ingenuity Expert Findings via ingenuity pathway analysis (https://digitalinsights.qiagen.com/products/qiagen-ipa).

#### Statistical analyses for significant metabolites and access to protein-metabolite interaction databases

Significant metabolites with differential levels were identified using R for the PTSD effect and were adjusted for coffee/energy drink intake, age and BMI. We used custom R functions/scripts and packages MetaboAnalystR^98^^,^^99^ and biomartR^100^ to access multiple databases of protein-metabolite interaction pathways and to search for the literature; we were thus able to link significant metabolites and proteins to relevant pathways and phenotypes. The drug-metabolite relations were identified using a repository of molecular interactions of ingenuity pathway analyses (IPA) (QIAGEN Redwood City, CA).

#### Statistical analysis of differentially methylated regions

Differentially methylated regions were identified using RnBeads (R package from Bioconductor), and Limma (R package). The outputs from RnBeads and Limma were overlapping with comparable significances (and rankings) of DMRs. Outputs from Limma were used for downstream integration since it was more straightforward in adjusting for confounders (age, the first three principal components of GWAS genotyping data for ancestry, BMI, cell composition, smoking status) as covariates while fitting the linear model.

#### Multi-omics integration and graphical representation of results

##### Clinical and multi-omics data integrations

MicroRNA protein regulator target interactions were identified using IPA. Protein-metabolome relations were created using metaboanalystR^98^^,^^99^ biomaRt^100^ and custom R functions. Some of the regulatory relations among *cis*-regulatory sites (differentially methylated regions or DMRs) and the corresponding proteins were identified based on the Encyclopedia of DNA Elements (ENCODE) v99-102 (https://www.encodeproject.org) and the literature accessed using biomaRt^100^ and custom R functions.

#### Networks, heatmaps, graphs and other forms of graphical representations

Custom R scripts/functions along with multiple R packages from the Comprehensive R Archive Network (https://cran.r-project.org/) and Bioconductor (www.bioconductor.org) such as ggplot2,^92^ WGCNA,^93^^,^^94^ ComplexHeatmap,^95^ circlize^96^ and igraph^97^ were used for graphical representation of analysis results. Networks were also rendered using Cytoscape (www.cytoscape.org), R and its plugins, igraph, and Gephi.^107^ Pathway and biological processes significantly associated with differentially changed proteins and metabolites were identified from Kyoto Encyclopedia of Genes and Genomes (KEGG),^108^ Reactome^109^ and GO^110^^,^^111^ databases accessed using custom R/python scripts/functions and biomaRt, as well as using KEGGscape, Bingo and Reactome FI plugins of Cytoscape, and NetworkAnalyst (https://www.networkanalyst.ca).

#### Genome-wide association study (GWAS) summary data and protein quantitative trait locus (pQTL)analysis

GWAS result tables were obtained from four large-scale publications: (1) MVP-PTSD genetic study consists of 186,689 participants for quantitative analysis and 214,408 (algorithmically defined 36,301 cases and 178,107 controls) total participants,^29^ (2) PGC-PTSD freeze-2 summary statistics data for European-ancestry participants (23,212 cases and 151,447 controls)^30^ and (3) PGC-MDD summary statistics data (59,851 cases and 113,154 controls),^90^ and (4) United Kingdom BioBank (UKBB) broad depression summary statistics data (113,769 cases and 208,811 controls).^91^

Genomic locations for genes coding PTSD associated proteins were obtained from ensemble database. The genetic variant with the lowest P-value in the GWAS data located in between the start and end location of the genes was identified for each gene. pQTL analysis was done by regressing normalized protein levels on additively coded genotype for common variants (minor allele frequency [MAF]>0.05). This analysis was done on SBC (n = 267) and FCC (n = 138) using MatrixEQTL R package.^101^ Only *cis*-regulated pQTL’s are considered (defined as variants located within 1Mb of the protein coding gene).

## Data Availability

• All the multi-omics and clinical datasets are available with permission through the SysBioCube, at https://sysbiocube-abcc.ncifcrf.gov. DOI is listed in the key resources table. • Custom manuscript-related code is located https://github.com/smuhie/multi-omics/blob/main/hPTSD_Transcriptome.R DOI is included in the key resources table. • Any additional information required to reanalyze the data reported in this work paper is available from the lead contact upon request.

## Supplemental information

Document S1. Figures S1–S8 and Tables S2–S4, S6, and S8

Table S1. Modular pathways with protein members, sorted by count, related to Figure 2 and the results subsections “consensus WGCNA network analyses” and “molecular indicators of PTSD” Member proteins of each module were filtered by significance level in the SBC Training cohort, whereby significance was determined using an FDR adjusted p value <0.1.

Table S5. MVP-GWAS variants associated with 37 significant proteins, related to Figure 4 and the results subsection “relevance of significant proteins in prior large-scale genetic studies” These variants have trending p value <5.0E-04.

Table S7. PGC-GWAS and pQTL variants and loci identified in the protein datasets of SBC and FCC male cohorts, related to Figure 4 and the results subsection “relevance of significant proteins in prior large-scale genetic studies”

Document S2. Article plus supplemental information
