# Supplementary material for: Molecular signatures of post-traumatic stress disorder in war-zone-exposed veteran and active-duty soldiers
Source: Cell Rep Med. 2023 May 16;4(5):101045. doi: 10.1016/j.xcrm.2023.101045 (PMC10213980; doi:10.1016/j.xcrm.2023.101045)
Supplement: Document S1. Figures S1–S8 and Tables S2–S4, S6, and S8 [file mmc1.pdf]

Cell Reports Medicine, Volume *4*

Supplemental information

Molecular signatures of post-traumatic stress disorder in war-zone-exposed veteran and active-duty soldiers

Seid Muhie, Aarti Gautam, Ruoting Yang, Burook Misganaw, Bernie J. Daigle Jr., Synthia H. Mellon, Janine D. Flory, Duna Abu-Amara, Inyoul Lee, Kai Wang, Ryan Rampersaud, PTSD Systems Biology Consortium, Leroy Hood, Rachel Yehuda, Charles R. Marmar, Owen M. Wolkowitz, Kerry J. Ressler, Francis J. Doyle III, Rasha Hammamieh, and Marti Jett

Table S2. Proteins associated with glycolytic and gluconeogenesis pathways (related to **Figures 4 & 5** and the results subsection ***"Metabolism and energy homeostasis"*** and ***"Metabolic dysregulations associated with PTSD")***

GeneName	Log2FC	*P* Value	Q Value	Pathway
ALDOA	-0.1971497	0.00629156	0.22204085	Gluconeogenesis
ALDOA	-0.1326427	0.0528327	0.16338075	Gluconeogenesis
GOT1	-0.1103362	0.03059705	0.30021916	Gluconeogenesis
GPT	-0.160358	0.05886906	0.37196953	gluconeogenesis
PGAM1	-0.283592	0.09033216	0.58662243	Gluconeogenesis
RBP4	-0.5639442	0.00062814	0.40826744	gluconeogenesis
RBP4	-0.2713165	0.01822838	0.90750904	gluconeogenesis
RBP4	-0.0927309	0.0122372	0.0564587	gluconeogenesis
ALDOA	-0.1205207	0.0743997	0.20185365	glycolysis
ENO1	0.2469892	0.00030339	0.00481815	glycolysis
ENO2	0.14344103	0.04839077	0.53175138	glycolysis
GAPDH	0.32914301	0.01098701	0.25167472	glycolysis
GPI	0.14348233	0.03305212	0.11304738	glycolysis
LDHB	0.1492963	0.01650351	0.38779645	glycolysis
MDH1	0.18087934	0.08265001	0.52141582	glycolysis
PGAM1	0.27561668	0.01285803	0.05746482	glycolysis
PGK1	0.05792659	0.0149839	0.06474833	glycolysis
PKM2	0.28506688	0.00038232	0.00554367	glycolysis
TPI1	0.08849913	0.08774704	0.22768171	glycolysis
ALDOA	-0.1205207	0.0743997	0.20185365	Glycolytic process
ENO1	0.2469892	0.00030339	0.00481815	Glycolytic process
ENO2	0.14344103	0.04839077	0.53175138	Glycolytic process
GAPDH	0.32914301	0.01098701	0.25167472	Glycolytic process
GPI	0.14348233	0.03305212	0.11304738	Glycolytic process
HK1	0.15960595	0.00019052	0.00388474	Glycolytic process
HK2	0.36458958	0.00053952	0.00670547	Glycolytic process
PGAM1	0.27561668	0.01285803	0.05746482	Glycolytic process

PGK1	0.05792659	0.0149839	0.06474833	Glycolytic process
TPI1	0.08849913	0.08774704	0.22768171	Glycolytic process

Table S3. Proteins associated with insulin resistance ((related to **Figures 4 & 5** and the results subsection ***"Metabolism and energy homeostasis"*** and ***"Metabolic dysregulations associated with PTSD")***

Gene Name	Log2FC	*P* Value	Q Value	Pathway
AKT1	0.09722531	0.01544152	0.06628678	Insulin resistance
IL6	0.12925092	0.07706642	0.22857198	Insulin resistance
INS	0.18178322	0.02503707	0.0928221	Insulin resistance
INSR	-0.1084194	0.02826253	0.10132582	Insulin resistance
MAPK8	0.15092143	0.01222405	0.0564587	Insulin resistance
PDPK1	0.42014017	6.73E-06	0.00058532	Insulin resistance
PIK3CA	0.07916264	0.01725798	0.07507222	Insulin resistance
PRKAA2	0.5148328	6.34E-05	0.00425568	Insulin resistance
PRKCB	0.36422865	5.10E-05	0.00165262	Insulin resistance
PRKCD	0.09221458	0.01061778	0.0507553	Insulin resistance
PRKCZ	0.18606991	0.05819511	0.19384389	Insulin resistance
PTPN1	0.21320595	0.00208641	0.02475235	Insulin resistance
PTPN11	0.25003138	0.00024074	0.00430357	Insulin resistance
RPS6KA3	0.18996703	0.00045353	0.00610158	Insulin resistance
STAT3	0.17212942	0.00123562	0.01211197	Insulin resistance
TNF	0.10577996	0.05477615	0.16470021	Insulin resistance
TNFRSF1A	-0.1872972	0.0073879	0.05165429	Insulin resistance
GSK3A	0.11196863	0.01738531	0.07537483	negative regulation of insulin receptor signaling pathway
IL1B	0.07736031	0.03081702	0.11296334	negative regulation of insulin receptor signaling pathway
PRKACA	0.26963996	0.00021529	0.00401365	negative regulation of insulin receptor signaling pathway
PRKCA	0.6450459	2.89E-05	0.00269026	negative regulation of insulin receptor signaling pathway

PRKCD	0.09221458	0.01061778	0.0507553	negative regulation of insulin receptor signaling pathway
PRKCZ	0.18606991	0.05819511	0.19384389	negative regulation of insulin receptor signaling pathway
PTPN1	0.21320595	0.00208641	0.02475235	negative regulation of insulin receptor signaling pathway
HIF1A	0.1264514	0.00015872	0.00339558	positive regulation of glycolysis
INS	0.18178322	0.02503707	0.0928221	positive regulation of glycolysis
INSR	-0.1084194	0.02826253	0.10132582	positive regulation of glycolysis

Table S4. Demographic compositions of cohorts and subgroups Data dictionary (related to **STAR Methods "*Cohort composition*")**

	SBC Training (N=218)	SBC Testing (N=82)	SBC Female (N=40)	FCC Female (N=21)	FCC Positive (N=47)	FCC Subthreshold (N=68)	FCC Negative (N=44)
**SEX**							
Male	218 (100%	82 (100%)	0 (0%)	0 (0%)	47(100)	68 (100%)	44 (100%)
Female	0 (0%)	0 (0%)	40 (100%)	21 (100%)	0 (0%)	0 (0%)	0 (0%)
**SELF-REPORTED RACE**							
Asian	9 (8.3%)	4 (3.7%)	0 (0%)	0 (0%)	0 (0%)	1 (1.5%)	1 (2.3%)
Black	28 (25.7%)	32 (29.4%)	15 (37.5%)	8 (38.1%)	2 (4.3%)	5 (7.4%)	7 (15.9%)
Hispanic	0 (0%)	2 (2.4%)	0 (0%)	3 (14.3%)	6 (13.0%)	13 (19.1%)	3 (6.8%)
White	49 (45.0%)	43 (39.4%)	13 (32.5%)	9 (42.9%)	36 (78.3%)	44 (64.7%)	31 (70.5%)
Other	7 (6.4%)	13 (11.9%)	12 (30.0%)	1 (4.8%)	3 (6.4%)	5 (7.4%)	2 (4.5%)
Missing	3 (2.8%)	4 (3.7%)					

	SBC Training (N=218)	SBC Testing (N=82)	SBC Female (N=40)	FCC Female (N=21)	FCC Positive (N=47)	FCC Subthreshold (N=68)	FCC Negative (N=44)
**Age**							
Mean (SD)	33.4 (8.34)	36.2 (9.48)	32.2 (6.41)	29.4 (7.63)	28.5 (5.54)	28.9 (5.60)	26.6 (4.53)
Median [Min, Max]	31.0 [20.0, 59.0]	34.0 [22.0, 59.0]	30.0 [24.0, 52.0]	27.0 [20.0, 46.0]	28.0 [20.0, 39.0]	28.0 [20.0, 43.0]	26.0 [20.0, 35.0]
**Smoking**							
**0**	172 (78.9%	48 (58.5%	36 (90.0%)	19 (90.5%	27 (57.4%)	38 (55.9%)	31 (70.5%)
**1**	38 (17.4%)	6 (7.3%)	4 (10.0%)	2 (9.5%)	20 (42.6%)	30 (44.1%)	11 (25%)
Missing	8 (3.7%)	28 (34.2%	0 (0%)	0 (0%)	0 (0%)	0 (0%)	2 (4.5%)
**Education**							
**1**	4 (1.8%)	1 (1.2%)	1 (2.5%)	0 (0%)	3 (6.4%)	5 (7.4%)	1 (2.3%)
**2**	59 (27.1%)	20 (24.4%	8 (20.0%)	8 (38.1%)	24 (51.1%)	40 (58.8%)	20 (45.5%)
**3**	61 (28.0%)	19 (23.2%	10 (25.0%)	6 (28.6%)	15 (31.9%)	14 (20.6%)	8 (18.2%)
**4**	66 (30.3%)	29 (35,4%	14 (35.0%)	6 (28.6%)	1 (2.1%)	5 (7.4%)	5 (9.6%)
**5**	27 (12.4%)	7 (8.5%)	7 (17.5%)	0 (0%)	0 (0%)	0 (0%)	0 (0%)
**6**	1 (0.5%)	3 (3.7%)	0 (0%)	0 (0%)	0 (0%)	0 (0%)	0 (0%)
Missing	0 (0%)	3 (3.7%)	0 (0%)	1 (4.8%)	4 (8,5%)	4 (5.9%)	10 (11.4%)
**BMI**							
Mean (SD)	28.8 (5.04)	30.1 (4.72)	26.8 (5.15)	26.0 (3.33)	27.3 (3.61)	28.4 (3.62)	27.3 (3.40)
Median [Min, Max]	28.0 [19.5, 49.9]	29.8 [21.5, 39.6]	25.9 [18.9, 38.0]	27.0 [21.0, 33.0]	28.0 [17.0, 34.0]	28.0 [21.0, 36.0]	27.0 [22.0, 35.0]

missing: data points not responded by participants (either skipped by mistake or not willing to answer); smoking: **0** – non-smoker, **1** – smoker; Education level (i.e., high school graduate = 12 years): **1** – Up to 12th grade; **2** – H.S. Diploma or GED (12 years); **3** – 2 yrs. college or A.A. Degree; **4** – 4 yrs. college or Bachelor's Degree; **5** – Masters Degree; **6** – Doctoral Degree

Table S6. Genes with significant variants identified in the Genome-Wide Associations study from the Million Veterans Program that were overlapping with PTSD-relevant modular networks of differentially methylated promoter regions (Related to **Figures 4** and the Results subsection **"Relevance of significant proteins in prior large-scale genetic studies")**

Size of PTSD-relevant module (# of genes with differentially methylated regions)	common with the 41 MVP genes	% overlap	Overlap enrichment (p-value)	significant pathway	pathway enrichment (q-value)
105	5	4.80%	1.19E-05	Axonogenesis	5.30E-03
119	10	8.40%	9.95E-13	directional locomotion (taxis)	1.10E-04
323	21	6.50%	2.61E-24	Nervous system development	2.90E-08

Table S8. Summary of blood tubes and samples for molecular assays (Related to **Table 1** and STAR Methods "***Blood draws***")

MARKERS	TUBE TYPE
miRNA, and Metabolomics	EDTA Lavender Top (LTT)
DNA-Methylation	PAX-gene (DNA)
Serum	SST, serum separating tube (tiger top tube)
Complete Blood Count (CBC)	EDTA Lavender Top (LTT)

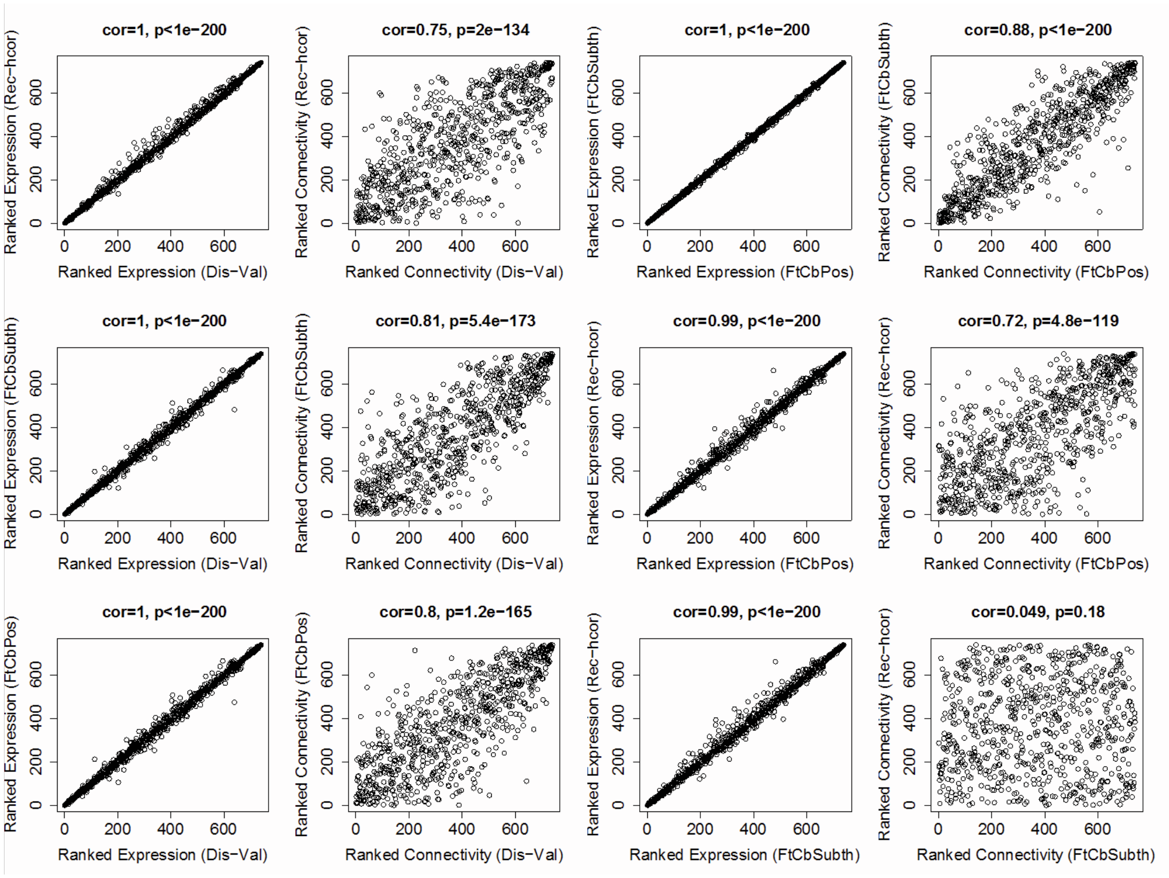

Fig. S1. QC output graphs to verify comparability of the four datasets: SBC Training SBC Testing, FCC Validation, FCC Subthreshold. Related to STAR METHODS (WGCNA for identification of modular networks). Key: SBC (Systems Biology Consortium), FCC (Fort Campbell Cohort)

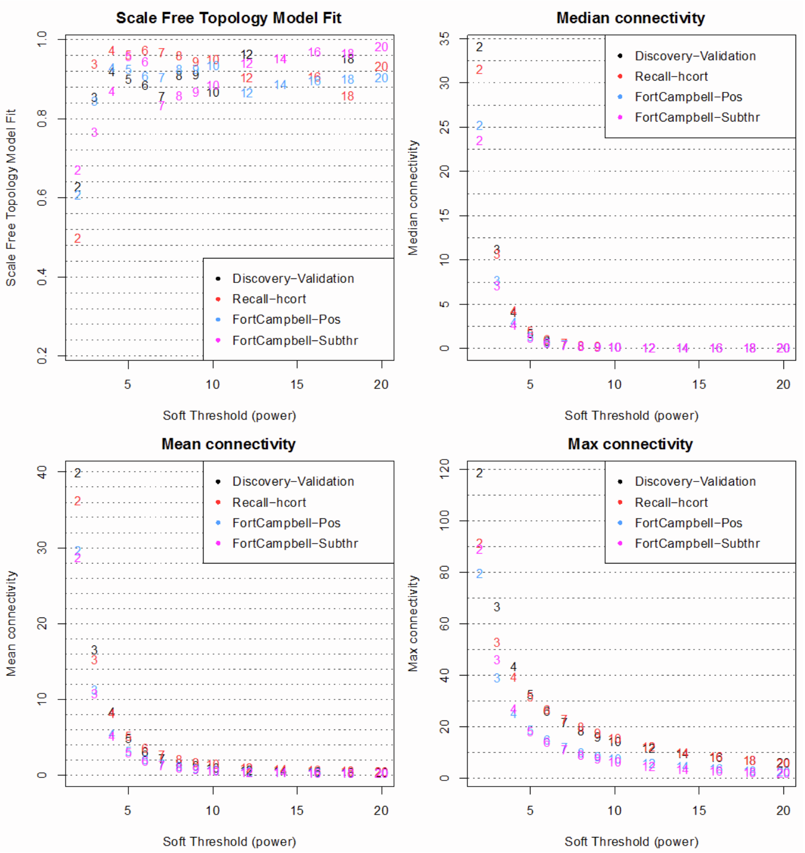

Fig. S2. Summary network indices as functions of soft thresholding power

Summary network indices (y-axes) were plotted as functions of the soft thresholding power (x-axes). Numbers in the plots indicate the corresponding soft thresholding powers. The plots indicate that approximate scale-free topology is attained around the soft-thresholding power of 4 for all of the sets. Because the summary connectivity measures decline steeply with increasing soft-thresholding power, it is advantageous to choose the lowest power that satisfies the approximate scale-free topology criterion. Related to STAR METHODS (WGCNA for identification of modular networks).

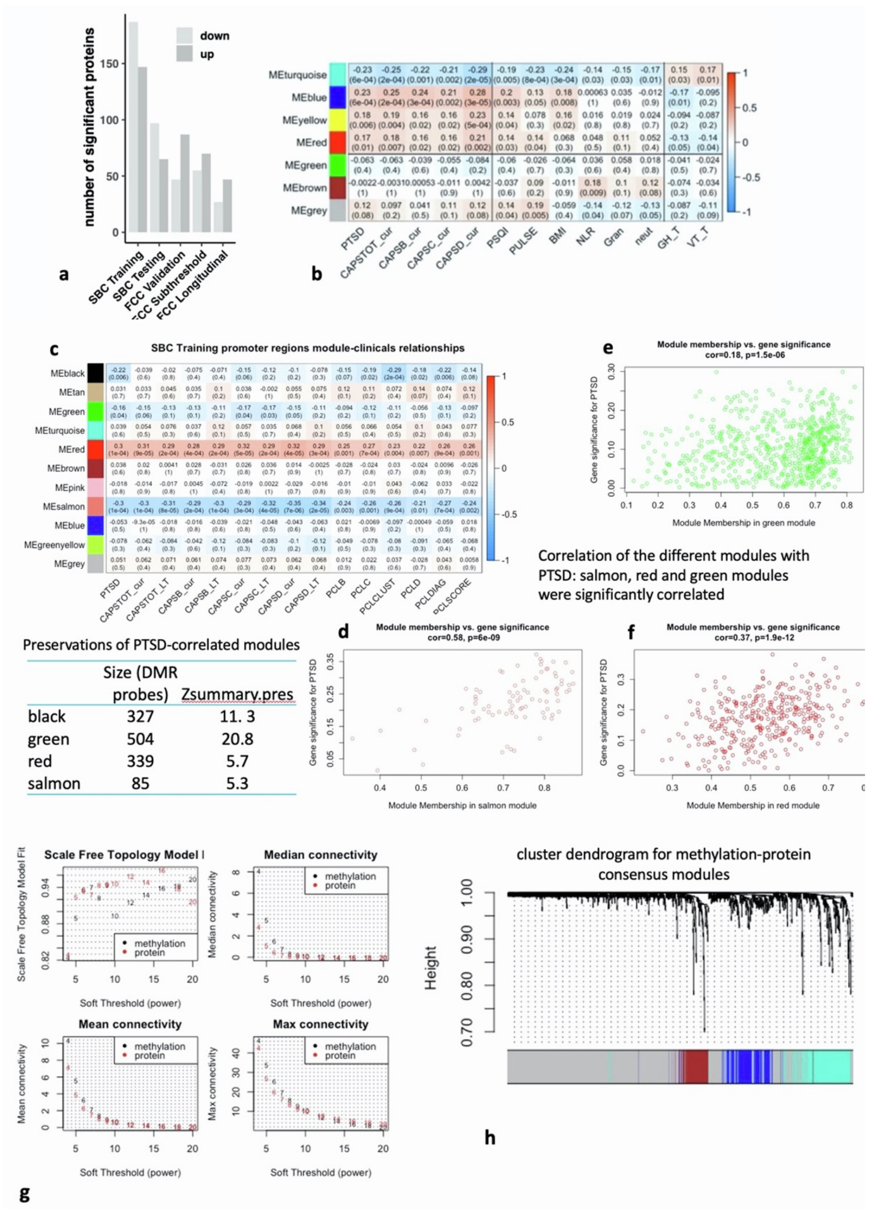

Fig. S3. Identification of protein co-expression modules associated with PTSD by consensus weighted gene co-expression network analysis (WGCNA)

(a) Number of significantly expressed proteins across subgroups of SBC and FCC cohorts **(b)** Correlation of modular networks with PTSD diagnostic and related clinical measurements. (**c-f**) Modules identified in the promoter regions of DNA methylation datasets, first identified in the discovery set of the 218 male veteran cohorts (**c**), and then their preservations and correlations with PTSD were checked (**c-f**). Four of the identified modules were correlated with PTSD total score, and two of these were significantly correlated with individual symptom clusters of PTSD (**c**), and were highly and moderately preserved in the other cohorts (preservations table). (**g**) Soft-power thresholding for identification of methylation-protein consensus networks. (**h**) Methylation (using datasets for probes located in the proximal promoter regions) and protein datasets (for the corresponding proteins) consensus modules (3 consensus modules were identified: red, blue, turquoise). Keys – BMI: body mass index; CAPSB_cur: current CAPS score, criterion B (re-experiencing); CAPSC_curr: current CAPS score, criterion C (avoidance); CAPSD_cur: current CAPS score, criterion D (negative alterations in cognition and mood); CAPSTOT_cur: current CAPS total score; GH_T: general health T-score; Gran: granulocyte; neut: neutrophil; NLR: node-like receptor; PSQI: Pittsburgh Sleep Quality Index; PTSD: positive for post-traumatic stress disorder; PULSE: pulse rate; Red: strong positive relationship; VT_T: vitality T-score); WGCNA: weighted gene correlation network analysis. Related to **Figure 2** and STAR METHODS (WGCNA for identification of modular networks).

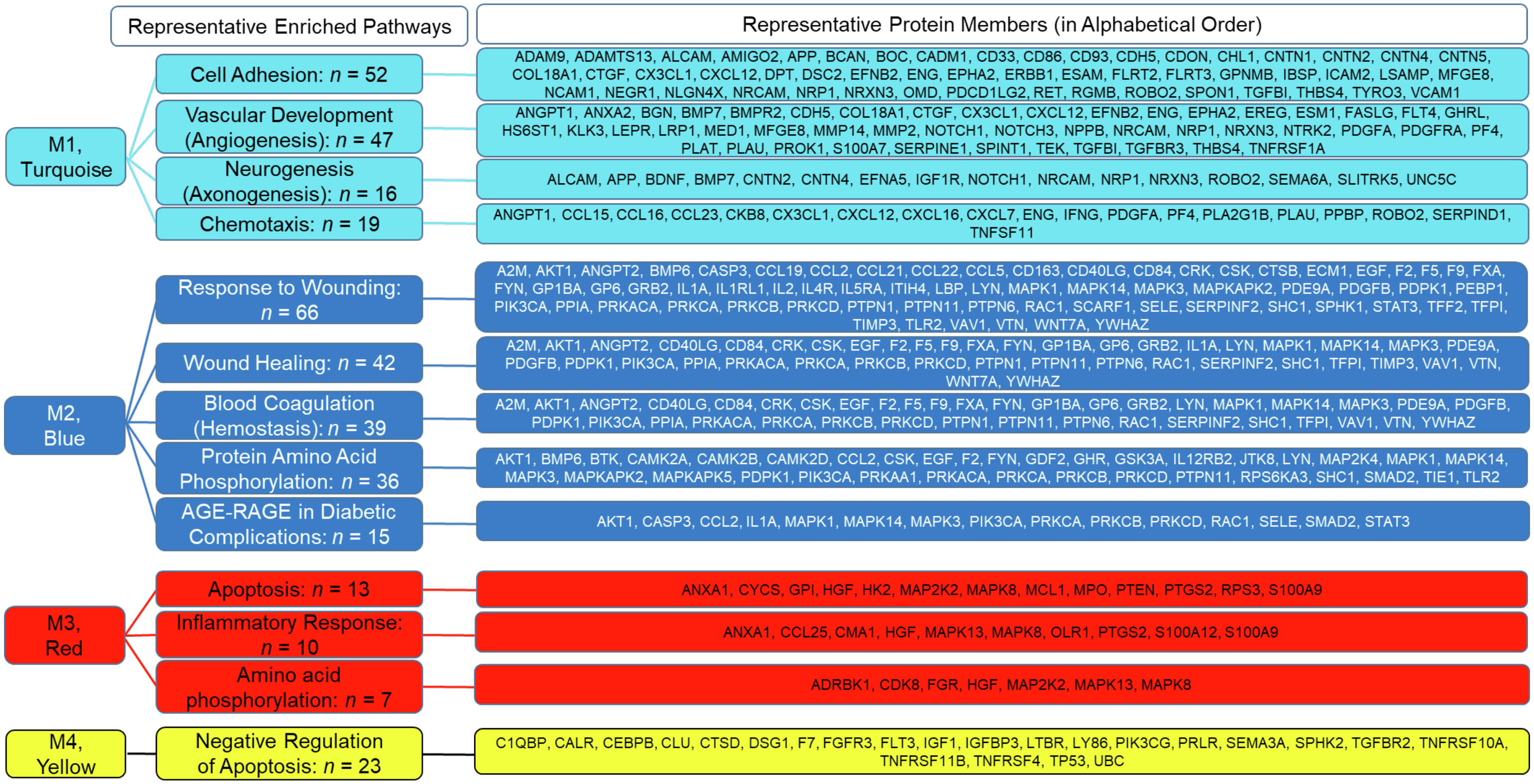

Fig S4. Member proteins within the 4 modules identified using protein data which were significantly correlated with PTSD (Complete data: Table S1)

Member proteins of each module were filtered by significance level in the SBC Training cohort, where significance was determined using an FDR adjusted *P* value < 0.1. Related to **Figure 2**. See **Table S1** for a complete list**.**

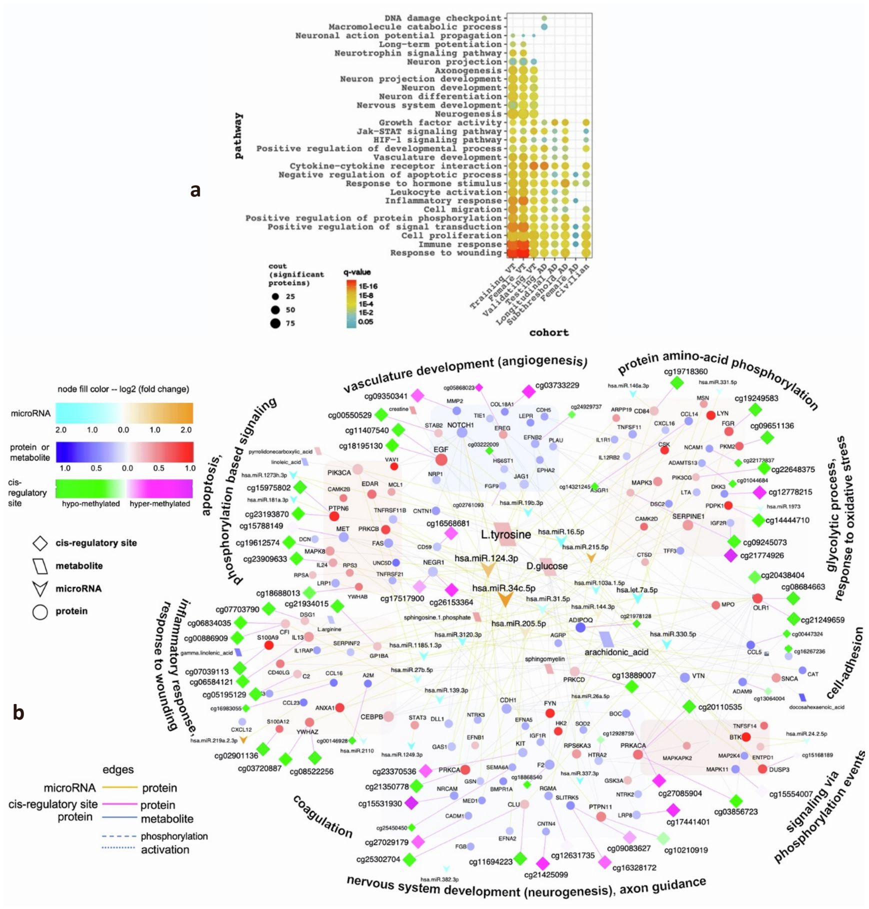

Fig. S5. a. Pathway enrichment analysis across all cohorts. Pathway or process enrichment analysis, FDR-corrected (PTSD cases vs controls) in the SBC Training, SBC Testing, FCC Validation, FCC Longitudinal, FCC Subthreshold, SBC Female and FCC Female cohorts. Civilians, 19 participants (10 PTSD+ and 9 PTSD-) which are recruited as part the Grady Trauma Project [1,2] were used for qualitative comparison with veteran and active duty cohorts. Related to **Figure 2**. **b. Multi-omics regulatory and functional network.** Network diagram shows regulatory and functional interconnections among molecular species significantly associated with the top pathways enriched in PTSD cases in the SBC Training, SBC Testing, and FCC Validation cohorts. This shows regulatory relationships between upstream epigenetic marks (miRs, and cis-regulatory sites – differentially methylated regions – DMRs) and downstream functional units (proteins and metabolites). Related to **Figure 4**. The upper gradient scale shows expression pattern of miRNA (blue – downregulated; orange – upregulated), protein or metabolite (blue – downregulated; red – upregulated; and cis-regulatory sites (green – hypomethylated; purple – hyper-methylated). Shapes within the network diagram correspond to the molecular species (diamond – cis-regulatory site; rhombus – metabolite; triangle – miR; circle – protein). Edges represent associations among molecular elements: yellow – miR-protein interaction; purple – cis-regulatory site protein-protein; blue – protein-metabolite; spaced dashed blue – phosphorylation; unspaced dashed blue – activation.

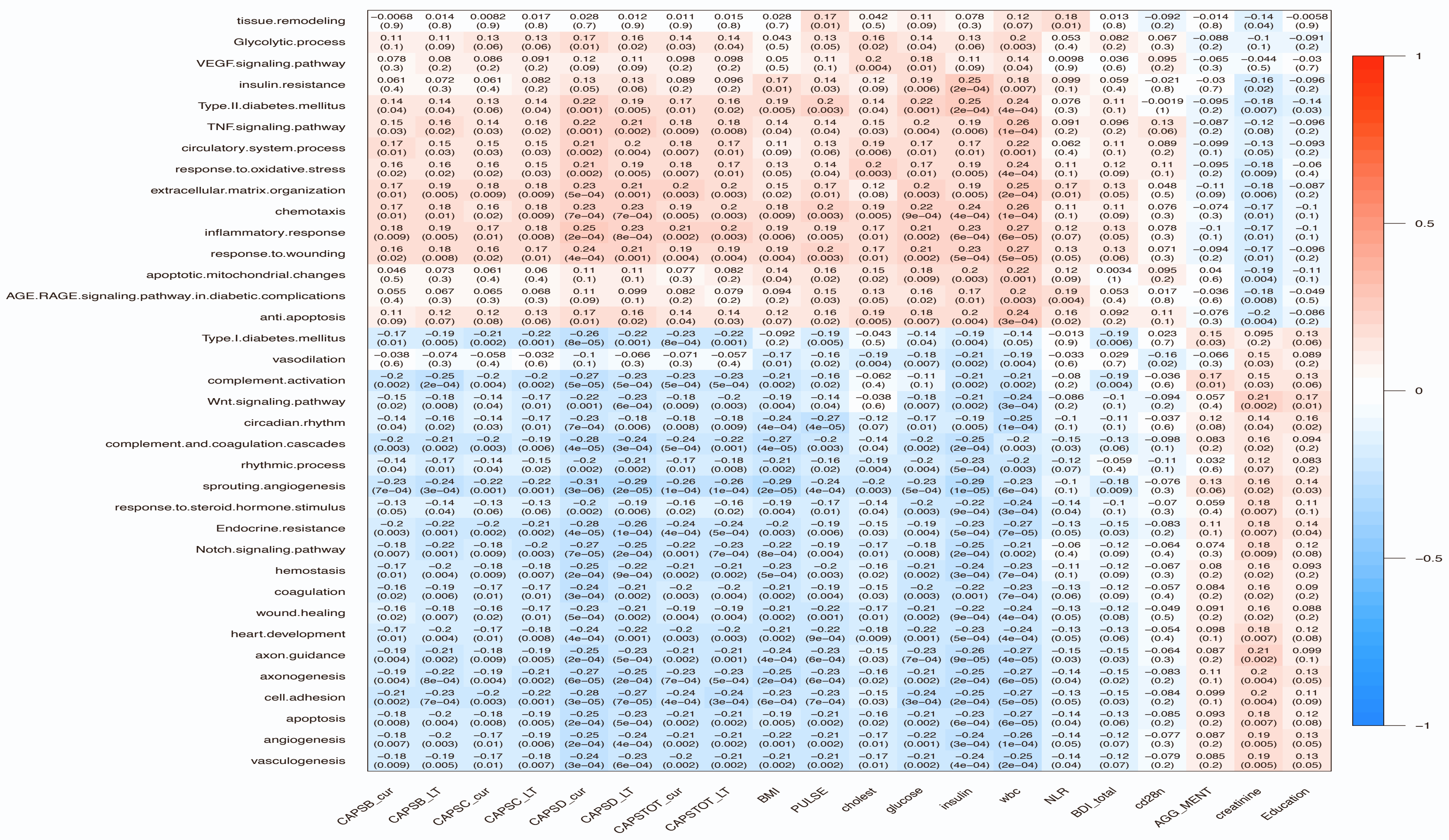

Fig. S6. Correlations among significant pathways and clinical variables

Values in parenthesis indicate significance of correlation; orange/red positively correlated, and blue negatively correlated. Related to **Figures 2 & 4**.

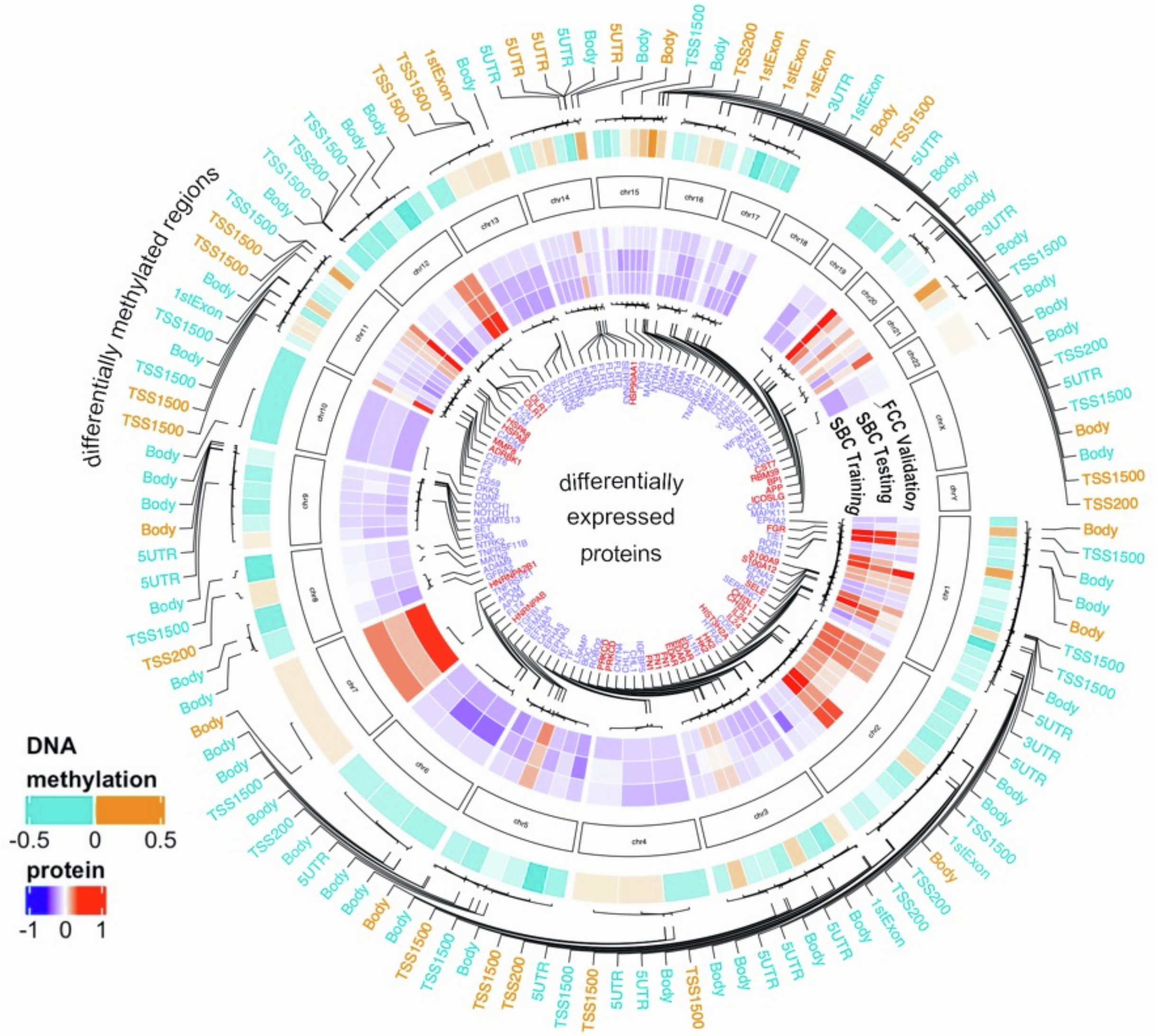

Fig. S7. Part of the multi-omics functional output

Potential cis-regulatory sites for proteins important in angiogenesis, axon guidance, and AGE-RAGE pathways (right). Angiogenesis and axon guidance were mainly associated with down-regulated proteins, whereas AGE-RAGE pathway was associated with up-regulated proteins. Related to **Figures 2 & 4**.

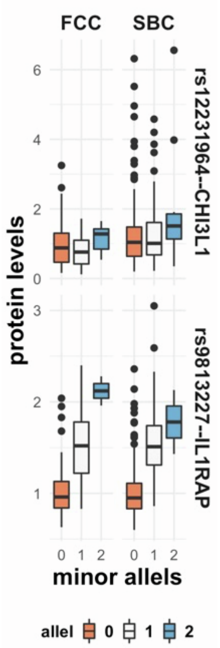

Fig S8. *cis*-acting pQTLs affecting serum levels of CHI3L1 and IL1RAP proteins. Protein expression data revealed an overlapping *cis*-expression pQTL-variant of CHI3L1 and IL1RAP that were associated with inflammatory response. Our analysis shows that genotype and disease phenotypes strongly associate with the serum inflammatory proteome in PTSD and identifies disease-associated pathways that may help to improve disease management in the future. Related to **Figure 4**.

References

1. Katrinli, S., Zheng, Y., Gautam, A., Hammamieh, R., Yang, R., Venkateswaran, S., Kilaru, V., Lori, A., Hinrichs, R., Powers, A., et al. (2021). PTSD is associated with increased DNA methylation across regions of HLA-DPB1 and SPATC1L. Brain Behav Immun *91*, 429-436. 10.1016/j.bbi.2020.10.023.
2. Lin, C., Michopoulos, V., Powers, A., Wingo, A.P., Schwartz, A., Bradley, B., Ressler, K.J., and Gillespie, C.F. (2018). Affect, inflammation, and health in urban at-risk civilians. J Psychiatr Res *104*, 24-31. 10.1016/j.jpsychires.2018.06.008.
